# Blue-shifted ancyromonad channelrhodopsins for multiplex optogenetics

**DOI:** 10.1101/2025.02.24.639930

**Published:** 2025-02-27

**Authors:** Elena G. Govorunova, Oleg A. Sineshchekov, Hai Li, Yueyang Gou, Hongmei Chen, Shuyuan Yang, Yumei Wang, Stephen Mitchell, Alyssa Palmateer, Leonid S. Brown, François St-Pierre, Mingshan Xue, John L. Spudich

**Affiliations:** 1 Center for Membrane Biology, Department of Biochemistry & Molecular Biology, The University of Texas Health Science Center at Houston McGovern Medical School, Houston, TX 77030, USA; 2 Department of Neuroscience, Baylor College of Medicine; Houston, TX 77030, USA; 3 The Cain Foundation Laboratories, Jan and Dan Duncan Neurological Research Institute at Texas Children’s Hospital; Houston, TX 77030, USA; 4 Department of Physics and Biophysics Interdepartmental Group, University of Guelph; Guelph, Ontario N1G 2W1, Canada; 5 Department of Biochemistry and Molecular Biology, Baylor College of Medicine, Houston, TX 77030, USA; 6 Systems, Synthetic, and Physical Biology Program, Rice University, Houston, TX 77005, USA; 7 Department of Electrical and Computer Engineering, Rice University, Houston, TX 7705, USA; 8 Department of Molecular and Human Genetics, Baylor College of Medicine, Houston, TX 77030, USA

## Abstract

Light-gated ion channels from protists (channelrhodopsins or ChRs) are optogenetic tools widely used for controlling neurons and cardiomyocytes. Multiplex optogenetic applications require spectrally separated molecules that must be found in nature, as they are difficult to engineer without disrupting channel function. Scanning numerous sequence databases, we identified three robust naturally blue-shifted ChRs from ancyromonads. They form a separate branch on the phylogenetic tree and contain residue motifs characteristic of anion ChRs (ACRs). However, only two conduct chloride, whereas the close *Nutomonas longa* homolog (peak absorption at ~440 nm) generates inward cation currents in mammalian cells under physiological conditions, significantly exceeding those by previously known tools. Measurements of transient absorption changes and pH titration of purified *Ancyromonas sigmoides* ACR (*Ans*ACR) combined with mutant analysis revealed the roles of the residues in the photoactive site. Both ancyromonad ACRs allowed optogenetic silencing of mouse cortical neurons in brain slices. *Ans*ACR expression in the cholinergic neurons enabled photoinhibition of pharyngeal muscle contraction in live worms. *Ans*ACR could be activated by near-infrared two-photon illumination, which is required to control specific neurons in thick tissue. Our results improved the mechanistic understanding of light-gated channel function and expanded the optogenetic toolkit.

**Impact statement:**

Ancyromonad channelrhodopsins advance our understanding of ionic selectivity and wavelength regulation in light-gated ion channels and also expand the toolkit for all-optical electrophysiology.

## Introduction

Channelrhodopsins (ChRs) are retinylidene proteins acting as photoreceptors that mediate photomotility in green flagellate algae (Sineshchekov, Jung, & Spudich, 2002) and are found in other protist lineages. The chromophore is attached via a retinylidene Schiff base (RSB) linkage to a conserved lysine residue in the 7^th^ transmembrane helix (TM7). Upon photoexcitation, ChRs generate passive ionic currents across the cell membrane and are used for optical control of excitable mammalian cells (optogenetics) (Deisseroth, 2021; Piatkevich & Boyden, 2023). The seven-transmembrane (7TM) domain is sufficient for channel activity; the role of the C-terminal domain comprising half of the polypeptide chain remains unclear. A considerable diversity within the ChR family suggests a convergent evolution of light-gated channel function (Govorunova, Sineshchekov, & Spudich, 2022). ChRs form dimers (Li et al., 2019; Volkov et al., 2017) or trimers (Morizumi et al., 2023; Tucker, Sridharan, Adesnik, & Brohawn, 2022), but their ionic conductance is intrinsic to individual protomers, unlike voltage- or ligand-gated channels, in which several protomers contribute to the channel pore. Anion channelrhodopsins (ACRs) generate photoinduced anion influx in mammalian cells; cation channelrhodopsins (CCRs) generate H^+^ and Na^+^ influx; and kalium channelrhodopsins (KCRs) generate K^+^ efflux (Govorunova, Sineshchekov, & Spudich, 2022; Govorunova, Sineshchekov, & Spudich, 2023). ACRs and KCRs are used for optogenetic neuronal inhibition, and CCRs are used for neuronal activation.

Increasingly popular all-optical electrophysiology, i.e., simultaneous perturbation and measurement of membrane potential using light-sensitive actuators and reporters, respectively in the same genetically defined cells (Hochbaum et al., 2014) requires spectrally non-overlapping optogenetic tools. Even the most red-shifted ChRs retain sufficient sensitivity to blue light due to their relatively wide spectral bandwidth (Govorunova et al., 2020; Oda et al., 2018). The development of red-light-absorbing genetically encoded fluorescent biosensors for monitoring neural activity (Sakamoto & Yokoyama, 2024) opened up the possibility of pairing them with blue-shifted ChRs. Molecular engineering yielded several blue-shifted ChR versions (Kato et al., 2015), but mutagenetic perturbations of the binding pocket frequently harm channel function. A complementary approach is exploring natural ChR diversity to search for molecules with desired biophysical properties optimized by evolution. Approximately ~1,000 ChR sequences are currently known, but a much smaller number has been functionally characterized (Govorunova, Sineshchekov, & Spudich, 2022).

Here, we identified and characterized three ChR variants from bacterivorous ancyromonad flagellates. Ancyromonads (also known as planomonads) that represent a distinct major clade near the most commonly inferred root of the eukaryote tree (Brown et al., 2018). We conducted automated and manual patch clamp analysis of photocurrents upon expression of ancyromonad ChR cDNAs in cultured mammalian cells and transient absorption changes in detergent-purified proteins. We show that two ancyromonad ChRs are anion-selective, but the third, the most blue-shifted, conducts metal cations. We expressed ancyromonad ACRs in mouse cortical pyramidal neurons and demonstrated photoinhibition of action potentials in acute brain slices. The nematode *Caenorhabditis elegans* is an attractive model for analyzing nervous system function by optogenetic manipulation (Bergs, Henss, Glock, Nagpal, & Gottschalk, 2022). C. *elegans* feeds on bacteria by rhythmic contractions and relaxations (pumping) of its pharynx. The cholinergic pharyngeal neurons, primarily the MC neurons, entrain the pharyngeal muscle rhythm (Trojanowski, Raizen, & Fang-Yen, 2016). Neuronal and muscular electrical activity leading to pharyngeal contractions can be monitored non-invasively by electropharyngeogram (EPG) recording (Raizen & Avery, 1994). We used this approach to demonstrate that a blue-shifted ancyromonad ACR enables efficient optogenetic inhibition of pharyngeal activity upon expression in the cholinergic neurons.

## Results

### Phylogeny and spectral sensitivity

**Figure 1.**
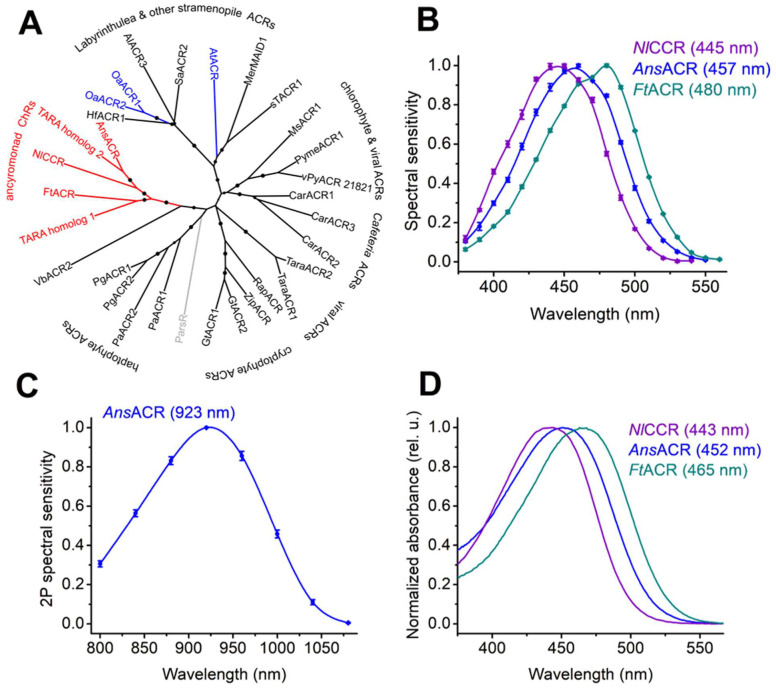
Phylogeny and spectral sensitivity of ancyromonad ChRs. (**A**) A maximum-likelihood phylogenetic tree of selected ChRs. The circles show bootstrap support from 40 to 100. (**B,C**) The photocurrent action spectra for 1P (B) and 2P (C) excitation. The symbols are the mean values; the error bars are SEM values (for 1P excitation, n = 14 cells for *Ans*ACR, and 9 cells each for *Ft*ACR and *Nl*CCR; for 2P excitation, n = 6 cells). (**D**) The absorption spectra of detergent-purified proteins. The online version of this article includes the following source data for Figure 1: Source data 1. Source data for the protein names and accession numbers used to construct the tree in (A); numerical values for the data shown in (B-D).

Our bioinformatic search identified ChR homologs in the ancyromonads *Ancyromonas sigmoides, Fabomonas tropica,* and *Nutomonas longa; Ancoracysta twista,* a predatory flagellate placed in the newly established supergroup Provora (Tikhonenkov et al., 2022); the diatom *Odontella aurita*; and *Paraphysoderma sedebokerense*, a chytrid-like fungus from the phylum Blastocladiomycota. Figure 1 – figure supplement 1 shows a protein alignment of their 7TM domains compared with *Gt*ACR1, the best-characterized ACR from the cryptophyte *Guillardia theta* (Govorunova, Sineshchekov, Liu, Janz, & Spudich, 2015; Li et al., 2019). All these sequences exhibit a non-carboxylate residue at the primary counterion (Asp85) position in TM3 of *Halobacterium salinarum* bacteriorhodopsin (BR), marked by the red arrow in Figure 1 – figure supplement 1, as found in all known ACRs. The 2^nd^ carboxylate in the photoactive site, contributed by TM7 and corresponding to Asp212 of BR, is conserved in all these homologs except the one from *N. longa*, in which it is replaced with Glu, and the one from *P. sedebokerense,* in which it is replaced with Gln (the blue arrow in in Figure 1 – figure supplement 1). Phylogenetic analysis placed the ancyromonad ChRs on a separate branch of the ACR tree together with their metagenomic homologs from the TARA Oceans database (Figure 1A). The *A. twista* and *O. aurita* homologs clustered with known stramenopile ACRs. The *P. sedebokerense* sequence showed only a distant homology to previously known ACRs.

We expressed cDNAs encoding the 7TM domains of all these homologs (except the metagenomic homolog) fused to a C-terminal mCherry tag in human embryonic kidney (HEK293) cells and recorded photocurrents using manual patch clamping. All three ancyromonad ChRs generated robust photocurrent and showed maximal sensitivity in the blue spectral range (Figure 1B). Based on their ionic selectivities (see the next section), we named the *A. sigmoides, F. tropica,* and *N. longa* homologs *Ans*ACR, *Ft*ACR, and *Nl*CCR, respectively. Optical manipulation of neuronal activity in dense tissue commonly relies on two-photon (2P) excitation based on the nearly simultaneous absorption of two infrared photons, equivalent to the absorption of one photon in the visible range (Emiliani et al., 2022). The following sections show that *Ans*ACR enables robust optogenetic inhibition of neuronal activity under one-photon (1P) illumination with visible light. We confirmed that *Ans*ACR can also be excited under 2P illumination, with a peak wavelength of ~920 nm (Figure 1C). Next, we expressed the constructs encoding ancyromonad ChRs in *Pichia.* We purified the proteins using a mild detergent yielding 0.25, 0.35, and 0.15 mg purified protein per L culture for *Ans*ACR, *Ft*ACR, and *Nl*CCR, respectively. The absorption spectra of the purified proteins were slightly blue-shifted from the respective photocurrent action spectra (Figure 1D), likely due to the presence of non-electrogenic *cis*-retinal-bound forms.

The spectral sensitivity of the *A. twista* and *O. aurita* homologs was in the blue-green range (Figure 1 – figure supplement 2, left). Partial replacement of Cl^−^ in the bath with non-permeable aspartate shifted reversal potential (V_r_) to more positive values, confirming their anion selectivity (Figure 1 – figure supplement 2, middle and right). We named them *At*ACR, *Oa*ACR1, and *Oa*ACR2. However, their photocurrents were smaller than those of the ancyromonad homologs or exhibited strong desensitization (reduction of photocurrents during illumination), so we did not characterize them in more detail. No photocurrents were detected in cells transfected with the *P. sedebokerense* homolog, although its expression and membrane targeting were evident from the tag fluorescence. Therefore, we named this protein *Pars*R, where R means “rhodopsin”.

### Characterization of ancyromonad ChRs by automated patch clamping

**Figure 2.**
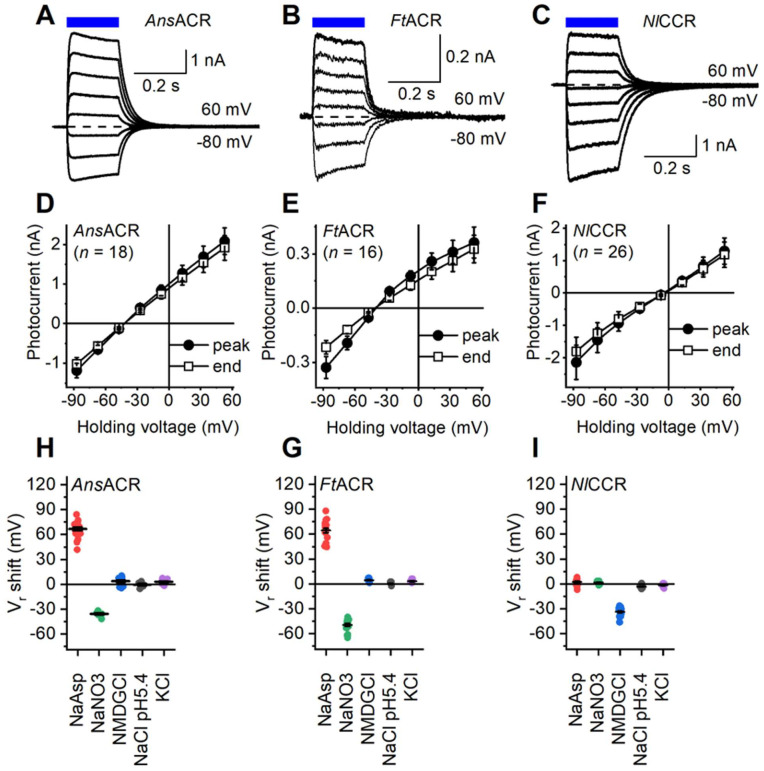
Characterization of ancyromonad ChRs by automated patch clamping. (**A**) Photocurrent traces recorded in response to 200-ms light pulses (470 nm) at voltages varied in 20-mV steps from −80 mV using the KF-based internal and NaCl-based external solutions. The dashed lines show the zero-current level. (**B**) The IV curves of the peak current (filled circles) and the current at the end of illumination (empty circles). The numbers in the parentheses are the numbers of cells sampled. (**C**) The V_r_ values in the indicated external solutions. The circles are the data from individual cells; the lines are the mean and sem values. The online version of this article includes the following source data for Figure 2: Source data 1. Source data for the numbers of cells sampled and numerical values shown in (B-C).

We used the fully automated planar patch clamp platform SyncroPatch 384 to characterize ancyromonad ChR photocurrents. This instrument uses KF-based internal and NaCl-based external solutions to promote gigaseal formation (Suppl. Table 1). Figure 2A-C shows photocurrent traces evoked by 200-ms light pulses. The SyncroPatch enables unbiased estimation of the photocurrent amplitude because the cells are drawn into the wells without considering their tag fluorescence, unlike manual patch clamp studies in which the experimenter selects the fluorescent cells. Figure 2 – figure supplement 1A, B shows that the mean *Ans*ACR photocurrent measured by the SyncroPatch was significantly larger than the mean *Ft*ACR photocurrent, and *Nl*CCR photocurrent was significantly larger than that of *Platymonas subcordiformis* channelrhodopsin 2 (*Ps*ChR2), a previously known excitatory optogenetic tool with a similar blue-shifted spectrum (Chen et al., 2022; Govorunova, Sineshchekov, Li, Janz, & Spudich, 2013).

The voltage dependencies of photocurrents (IV curves) at the peak time and the end of the light pulse (Figure 2D-F) showed the same reversal potential (V_r_), indicating no change in the relative permeability during illumination. *Ans*ACR and *Ft*ACR showed similarly negative V_r_ values: −43 ± 3 and −41 ± 1 mV, respectively (mean ± sem, n = 22 and 16 cells), suggesting higher relative permeability to Cl^−^ than F−, as earlier found in *Gt*ACRs by manual patch clamping (Govorunova et al., 2015). Unexpectedly, the *Nl*CCR photocurrents reversed near 0 mV (−1 ± 1 mV, mean ± sem, n = 27 cells). One reason for this behavior could be equal permeabilities of this ChR to Cl^−^ and F^−^. However, when Cl^−^ in the external solution was partially replaced with bulky, non-permeable aspartate, only *Ans*ACR and *Ft*ACR showed substantial V_r_ shifts to more positive values (Figure 2G-I, red symbols), which revealed their permeability to Cl^−^, but no such shift was detected in *Nl*CCR. Control experiments conducted by manual patch clamping with the Cl^−^-based pipette solution confirmed these observations (Figure 2 – figure supplement 2). Upon replacing Cl^−^ with NO_3_^−^, *Ans*ACR and *Ft*ACR, but not *Nl*CCR, showed V_r_ shifts to more negative values (Figure 2G-I, green), as did *Gt*ACRs examined by manual patch clamping (Govorunova et al., 2015). *Ft*ACR exhibited a larger V_r_ shift in NO_3_^−^ than *Ans*ACR (p = 2.6E10-5 by the two-tailed Mann-Whitney test). Replacing Na^+^ with N-methyl-D-gluconate (NMDG^+^) resulted in a negative V_r_ shift in *Nl*CCR but not the other tested variants (Figure 2G-I, blue symbols), suggesting that *Nl*CCR is permeable to Na^+^. Figure 2 – figure supplement 1C, D compares *Nl*CCR with the typical cation-selective *C. reinhardtii* channelrhodopsin 2 (*Cr*ChR2) tested by the same assay. Acidifying the external solution to pH 5.4 or replacing Na^+^ with K^+^ did not affect the V_r_ of any ancyromonad ChR (Figure 2G-I, grey and violet). We conclude that only *Ans*ACR and *Ft*ACR are anion-selective, but *Nl*CCR conducts monovalent metal cations despite its sequence homology to the other two variants.

### Channel gating and photochemical transitions under single-turnover conditions

**Figure 3.**
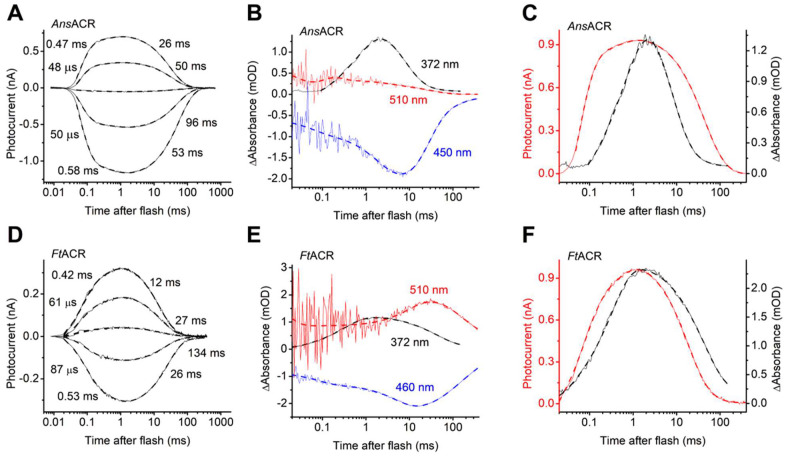
Photocurrent and transient absorption changes under single-turnover conditions in *Ans*ACR and *Ft*ACR. (**A, D**) Photocurrent traces of *Ans*ACR (A) and *Ft*ACR (D) evoked by 6-ns laser flashes recorded by manual patch clamping at the holding voltages increased in 30-mV steps from −60 mV. (**B, E**) Transient absorption changes recorded at the indicated wavelengths from detergent-purified proteins. (**C, F**) Comparison of the photocurrent kinetics (red, left axis) and the M intermediate kinetics (black, left axis). In all panels, the thin, solid lines are experimental recordings, and the thick, dashed lines are multiexponential approximations. The numbers are the τ values of the individual kinetic components.

Photocurrents evoked by continuous light pulses do not accurately reflect channel kinetics because different ChR molecules absorb photons at different times. Further complications result from photon absorption by photocycle intermediates. We conducted manual patch clamp recordings upon 6-ns laser flash excitation to analyze channel gating. *Ans*ACR and *Ft*ACR photocurrents exhibited biphasic rise and decay (Figure 3A, D), as the earlier characterized *Gt*ACR1 (Sineshchekov, Govorunova, Li, & Spudich, 2015). The IV curves of all kinetic components revealed the same V_r_ values (Figure 3 – figure supplement 1A, B), meaning no ion selectivity changes occur during the single-turnover photocycle. Next, we analyzed transient absorption changes in detergent-purified *Ans*ACR and *Ft*ACR. In contrast to *Gt*ACR1, we could not find a clear indication of the accumulation of a blue-shifted L intermediate. After an initial (unresolved) decay of a K-like intermediate, red-shifted absorbance temporally increased in both ancyromonad ACRs, reaching a maximum at 100-200 μs (Figure 3B, E, red), i.e., in the time domain of fast channel opening. This red-shifted intermediate could be a long-lived K or an unusual red-shifted L. In *Ft*ACR, an additional slower increase in the red-shifted absorbance likely reflected the formation of an O-like intermediate (Fig. 3E, red). Accumulation of the M intermediate absorbing in the UV range was observed 10 (*Ans*ACR) or 2 times (*Ft*ACR) slower than channel opening (Figure 3C, F), in contrast to *Gt*ACR1, in which it was 50 times slower (Sineshchekov et al., 2015). Unlike *Gt*ACR1, no temporal correlation was found between M formation and fast channel closing and between M decay and slow channel closing in ancyromonad ACRs.

**Figure 4.**
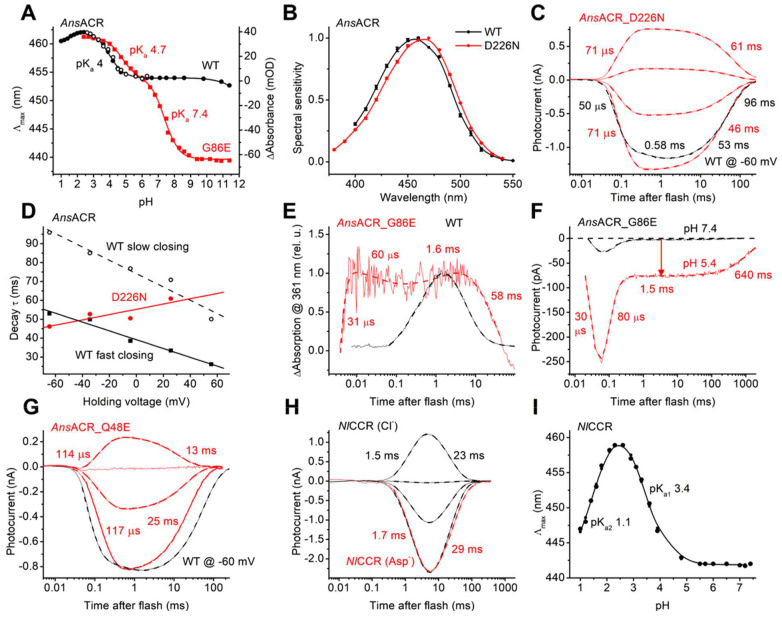
Characterization of the wild-type proteins and *Ans*ACR mutants. (**A**) pH titration of λ_max_ (black filled circles, left axis) and maximal absorption changes (black empty circles, right axis) in wild-type *Ans*ACR, and λ_max_ in *Ans*ACR_G86E mutant (red, left axis). (**B**) The photocurrent action spectrum of the *Ans*ACR_D226N mutant (red) compared to the WT (black). The symbols are the mean values, and the error bars are SEM values (n = 8 cells). (**C, G**) Photocurrent traces of *Ans*ACR_D226N mutant (C) and *Ans*ACR_Q48E mutant (G) evoked by 6-ns laser flashes recorded by manual patch clamping at the holding voltages increased in 30-mV steps from −60 mV. The wild-type photocurrent trace recorded at −60 mV is shown in black for comparison. The thin lines are experimental recordings, and the thick dashed lines are multiexponential approximations. The numbers are the τ values of the individual kinetic components. (**D**) The voltage dependence of the decay components τ in the *Ans*ACR_D226N mutant (red) and the WT (black). (**E**) Transient absorption changes monitored at the wavelength of the M intermediate absorption in the *Ans*ACR_G86E mutant (red) compared to the WT (black). (**F**) Laser-flash-induced photocurrents of the *Ans*ACR_G86E mutant recorded at the external pH 7.4 (black) and 5.4 (red). The arrow shows the increase in the channel current upon acidification. (**H**) Laser-flash evoked photocurrent traces of *Nl*CCR recorded at the holding voltages increased in 30-mV steps from −60 mV using the Cl^−^-(black) or Asp^−^-based (red) bath solution. (**I**) pH titration of detergent-purified *Nl*CCR. The online version of this article includes the following source data for Figure 4: Source data 1. Source data for the numerical values shown in (A, B, D, and I).

A pH titration of the peak wavelength (λ _max_) (Figure 4A, black filled circles, left axis) or maximal absorption changes (Figure 4A, black empty circles, right axis) in the wild-type *Ans*ACR revealed the main transition with a pK_a_ 4. The 4-nm redshift of λ _max_ observed in the wild type (WT) upon acidification corresponded to the ~8 nm redshift of the photocurrent action spectrum of the *Ans*ACR_D226N mutant relative to the WT (Figure 4B). Therefore, we conclude that this spectral transition in the WT reflects the titration of the counterion Asp226. Photocurrent in the *Ans*ACR_D226N mutant was not much affected, but its rise and decay became monophasic (Figure 4C). Furthermore, the monophasic decay in this mutant accelerated upon depolarization, in contrast to both decay phases in the WT (Figure 4D.

All ACRs have a non-carboxylate residue in the position of Asp85 in BR (Figure 1 – figure supplement 1, red arrow). Mutagenetic introduction of Glu in this position in *Ans*ACR (the G86E mutation) caused a new pH-induced spectral transition with a pK_a_ of 7.4 and a shift of the pK_a_ in the acidic range from 4.0 to 4.7 (Figure 4A, red). This mutation also led to the appearance of an extremely fast (τ 31 μs) M-like UV-absorbing intermediate (Figure 4E, red). Most importantly, this mutation eliminated channel current at neutral pH and led to the appearance of a fast current. The rise and decay τ of the latter corresponded to the rise and decay τ of fast M formation (compare Figures. 4E and F). Acidification of the bath to pH 5.4 recovered channel current (Figure 4F, red), the kinetics of which differed from that in the WT (Figure 3A). The full current trace could be deconvoluted into four components with τ 30 μs, 80 μs, 1.5 ms, and 640 ms, revealing that the mutation slowed channel closing 6-fold. Replacement of Cl^−^ with Asp^−^ caused a~40-mV shift of the channel current’s V_r_ (Figure 3 – figure supplement 1C), indicating that the mutant channel remained Cl^−^ selective. Acidification increased the amplitude of the fast current ~10-fold (Figure 4F) and shifted its V_r_ ~100 mV (Figure 3 – figure supplement 1D), as expected of passive proton transport. The number of charges transferred during the fast peak current was >2,000 times smaller than during the channel opening, from which we concluded that the fast current reflects the movement of the RSB proton. Similar τ values of the fast current decay and the decay of fast M formation (Figures 4E and F) corroborate this conclusion. These results confirm that the absence of a negative charge at BR’s primary acceptor site is the ultimate condition for anion channel function. The glutamate in the middle of TM2 corresponding to Glu68 of *Gt*ACR1 is conserved in most ACRs, including *Ft*ACR, but replaced with Gln in *Ans*ACR (Figure 1 – figure supplement 1, black arrow). The Q48E mutation in AnsACR accelerated channel closing and slowed channel opening, making the photocurrent rise and decay monophasic (Figure 4G, Figure 3 – figure supplement 1E).

In contrast to *Natronomonas pharaonis* halorhodopsin (Varo, Brown, Needleman, & Lanyi, 1996), no blue spectral shift was detected in detergent-purified *Ans*ACR upon an increase in the Cl^−^ concentration (Figure 4 – figure supplement 4A), which argued against Cl^−^ binding in the RSB region at neutral pH. Strong alkalization caused simultaneous depletion of absorption in the visible range, the appearance of the M-like states (at 368 nm in the wild-type *Ans*ACR and 355 nm in the *Ans*ACR_G86E mutant), and a substantial absorption increase at 297 nm, reflecting deprotonation of the RSB and a strong perturbation of the protein band (Figure 4 – figure supplement 1B). Analysis of the pH dependence of three parameters (absorption depletion in the visible range, absorption rise in the M-like states’ range, and absorption rise at 297 nm) yielded similar pK_a_ values, which were 11.1 and 10.7 for the WT and G86E, respectively (Figure 4 – figure supplement 1C). These high pK_a_ values may explain high photostability of this protein, as hundreds of laser flashes did not cause its measurable bleaching.

In contrast to *Ans*ACR and *Ft*ACR, only two exponentials were sufficient to fit *Nl*CCR laser-flash-evoked photocurrents, both in Cl^−^ and Asp^−^ bath solutions (Figure 4H, Figure 3 – figure supplement 1F). We conducted pH titration of its stationary absorption (Figure 4I). From analogy with *Ans*ACR, we interpret the spectral transition with pK_a1_ 3.4 as protonation of the residue corresponding to Asp212 in BR, which is Glu233 in *Nl*CCR (Figure 1 – figure supplement 1, blue arrow). Upon further acidification, *Nl*CCR exhibited a second large transition to shorter wavelengths with pK_a2_ 1.1, only slightly presented in ancyromonad ACRs. A similar transition was observed in haloarchaeal rhodopsins and explained by Cl^−^ binding in the photoactive site (Shimono, Kitami, Iwamoto, & Kamo, 2000).

### The retinal-binding pocket and color tuning

**Figure 5.**
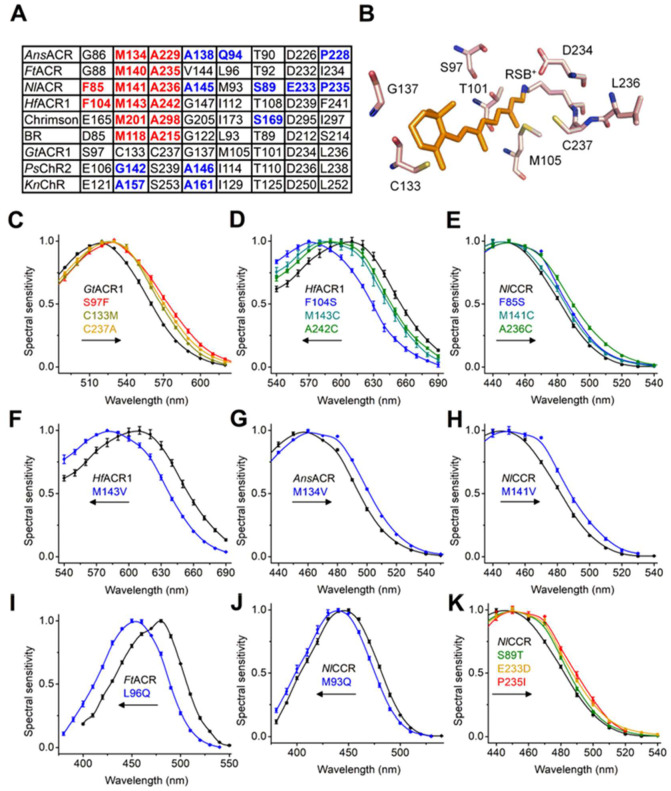
Color tuning of ancyromonad ChRs. (**A**) Amino acid residues of the retinal-binding pocket tested by mutagenesis in this study. (**B**) The corresponding residues in the *Gt*ACR1 structure (6edq). (**C-K**) The photocurrent action spectra of the indicated mutants compared to the respective WTs. The symbols are the mean values, the error bars are SEM values. The online version of this article includes the following source data for Figure 5: Source data 1. Source data for the numbers of cells sampled and numerical values shown in (C-K).

The λ_max_ of rhodopsins is regulated by the retinal chromophore geometry and steric and electrostatic interactions of the chromophore with amino acid residues of the retinal-binding pocket (Hoffmann et al., 2006; Karasuyama, Inoue, Nakamura, Kandori, & Takeuchi, 2018). Surprisingly, *Nl*CCR, the most blue-shifted among ancyromonad ChRs, features three residues typical of red-shifted microbial rhodopsins (Figure 5A). The first is Phe at the primary counterion position (Asp85 in BR), also found in RubyACRs from Labyrinthulea, the most red-shifted ChRs so far identified (Govorunova et al., 2020). The residues homologous to BR’s Met118 near the β-ionone ring and Ala215 preceding the RSB lysine in the polypeptide chain are responsible for red-shifted absorption in many microbial rhodopsins (Engqvist et al., 2015; Oda et al., 2018; Oppermann et al., 2024; Shimono, Iwamoto, Sumi, & Kamo, 2000) but are conserved in all three blue-absorbing ancyromonad ChRs. In *Gt*ACR1 (λ_max_ 515 nm, (Govorunova et al., 2015; Sineshchekov, Li, Govorunova, & Spudich, 2016)), the only ACR with published atomic structures (Kim et al., 2018; Li et al., 2019), the corresponding residues are Ser97, Cys133, and Cys237 (Figure 5B). As expected, the S97F, C133M, and C237A mutations red-shifted the *Gt*ACR1 spectrum (Figure 5C). The opposite F104S, M143C, and A242C mutations at the corresponding sites in the RubyACR from *Hondaea fermentalgiana* (*Hf*ACR1) caused large blue spectral shifts (Figure 5D). However, the corresponding mutations F85S, M141C, and A236C red-shifted the *Nl*CCR spectrum (Figure 5E). The same paradoxical behavior was observed upon mutation of the Met118 homolog to Val, which blue-shifted the *Hf*ACR1 spectrum but red-shifted the ancyromonad ChR spectra (Figure 5F-H). Replacement of the Ala215 homolog with Cys or Ser did not change the *Ans*ACR and *Ft*ACR spectra (Figure 5 – figure supplement 1A, B).

In blue-shifted ChRs such as *Platymonas subcordiformis* channelrhodopsin 2 (*Ps*ChR2) (Govorunova et al., 2013) and *Klebsormidium nitens* channelrhodopsin (*Kn*ChR) (Tashiro et al., 2021), the position of BR’s Met118 is occupied by Gly or Ala (Figure 5A), which, together with the Ala four residues downstream (BR’s Gly122), rotates the β-ionone ring out of the plane of the polyene chain, shrinking the *p*-conjugation and blue-shifting the spectrum (Wang et al., 2024). This Ala is conserved in *Ans*ACR and *Nl*CCR (Figure 5A), but the *Ans*ACR_M134A/G and *Nl*CCR_M141A/G mutations did not change the spectra, nor did the *Ft*ACR_M140G_V144A mutation (Figure 5 – figure supplement 1A-C). These observations suggest that the residue geometry and/or interactions in the retinal-binding pocket in ancyromonad ChRs differ from the earlier studied microbial rhodopsins.

The residue position corresponding to BR’s Leu93 is the color switch between blue- and green-absorbing proteorhodopsins (BPRs and GPRs, (Man et al., 2003)). *Ans*ACR exhibits a Gln residue in this position, as do BPRs, but *Ft*ACR has Leu, as do GPRs, and *Nl*CCR has Met (Figure 5A). The *Ft*ACR_L96Q and *Nl*CCR_M93Q mutations blue-shifted the spectra 20 and 7 nm, respectively (Figure 5I, J), indicating that this residue position contributes to color tuning in ancyromonad ACRs. *Nl*CCR, the most blue-shifted among ancyromonad ChRs, differs from *Ans*ACR and *Ft*ACR at the positions corresponding to Ser89 and Glu233 (*Nl*CCR numbering), and from *Ft*ACR, also at the position of Pro235 (the corresponding residues in *Gt*ACR1 are Thr101, Asp234, and Leu236, Figure 5A). The S89T, E233D, and P235I mutations red-shifted the *Nl*CCR spectrum (Figure 5K), indicating that these three positions contribute to the blue shift of wild-type *Nl*CCR compared to *Ans*ACR and *Ft*ACR. However, the T90S mutation did not change the *Ans*ACR spectrum, and the D226E mutation and a combination of the two mutations caused red spectral shifts in *Ans*ACR (Figure 5 – figure supplement 1D).

### Optogenetic inhibition of cortical neurons in mouse brain slices

**Figure 6.**
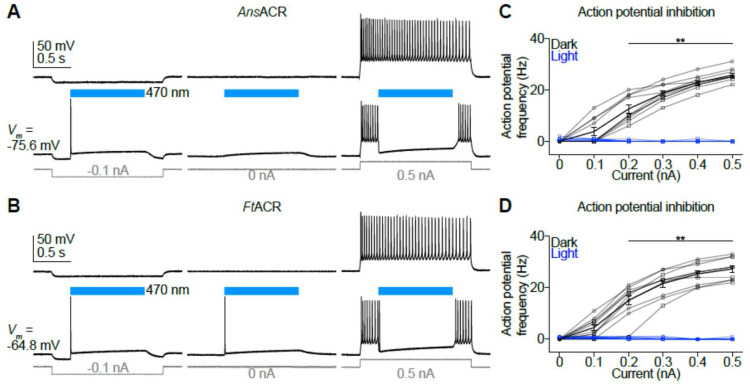
Photoactivation of *Ans*ACR and *Ft*ACR inhibits the action potentials of mouse cortical neurons. (**A, B**) Representative membrane voltage traces of neurons expressing *Ans*ACR (A) and *Ft*ACR (B) in response to −0.1 nA (left), 0 nA (middle), and 0.5 nA (right) injections without (top) and with (bottom) 470 nm light pulses (power density of 38.7 mW mm^−2^). (**C, D**) The frequencies of action potentials evoked by different current injections with (blue) and without (black) photoactivation of *Ans*ACR (C) and *Ft*ACR (D). For all panels, data points from male mice are indicated by squares and female mice by circles. One male and one female mouse were used for each of the *Ans*ACR and *Ft*ACR experiments. Data are mean ± sem. **, p ≤ 0.01 for 0.2-0.5 nA by the multiple Wilcoxon matched-pairs signed rank test with Benjamini, Krieger, and Yekutieli’s corrections. The online version of this article includes the following source data for Figure 6: Source data 1. Source data for the numerical values shown in (C, D).

To test the silencing efficiencies of *Ans*ACR and *Ft*ACR in mice, we selectively expressed their 7TM domains fused with EYFP in the layer 2/3 pyramidal neurons of the somatosensory cortex by *in utero* electroporation at embryonic day 15. Bright fluorescence was observed in the somata, dendrites, and axons, indicating high-level *Ans*ACR-EYFP and *Ft*ACR-EYFP expression (Figure 6 – figure supplement 1A). We prepared acute brain slices from 4-6-week-old mice and performed whole-cell current clamp recordings from *Ans*ACR- or *Ft*ACR-expressing neurons (for solution compositions, see Material and Methods). *Ans*ACR- and *Ft*ACR-expressing neurons showed the resting membrane potential, input resistance, and capacitance values within the range typical of untransfected cortical neurons (Figure 6 – figure supplement 1B), suggesting that expression of these ACRs induced negligible cytotoxicity. When positive currents were injected into the somata to excite neurons, photoactivation of *Ans*ACR and *Ft*ACR suppressed the current-evoked action potentials, demonstrating the potency of these proteins as optogenetic silencers of mouse cortical neurons (Figure 6). We also observed that at rest or when a small negative current (e.g., −0.1 nA) was injected, the neurons could generate a single action potential at the beginning of photostimulation (Figure 6A, B), similar to *Gt*ACR-expressing neurons (Mahn et al., 2018; Messier, Chen, Cai, & Xue, 2018).

Earlier studies have shown that photoactivation of *Gt*ACRs induces axonal depolarization and synaptic transmission in some ACR^+^ neurons owing to the high intracellular Cl^−^ concentration at the axons (Mahn et al., 2018; Messier et al., 2018). Indeed, when we recorded from ACR^−^ neurons in the electroporated cortical region, we found that photoactivation of either *Ans*ACR- or *Ft*ACR-induced excitatory post-synaptic currents (Figure 6 – figure supplement 1C, D) similar to other tested light-gated Cl^−^ channels.

### Optogenetic inhibition of pharyngeal function in live C. elegans

**Figure 7.**
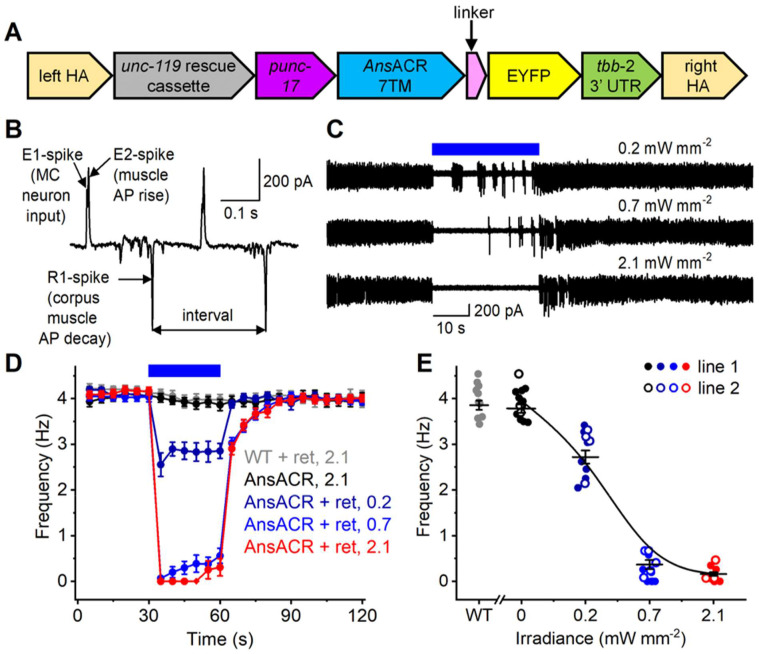
Photoinhibition of pharyngeal pumping in live *C. elegans* expressing *Ans*ACR in the cholinergic neurons. (**A**) A scheme of the genetic construct for *Ans*ACR expression in the cholinergic neurons. HA, homology arms. (**B**) A zoomed-in section of an EPG recording. AP, action potential; TB, terminal bulb. The double-headed arrow shows the interval between two successive R1 spikes used to calculate pharyngeal pumping frequency. (**C**) Electropharyngeogram recordings from an *Ans*ACR-expressing worm illuminated with 470 nm light at the indicated irradiances. The blue bar shows the duration of illumination. (**D**) The frequency of the pharyngeal pumping calculated from recordings as shown in A. The symbols are the mean values, and the error bars are the SEM values (n = 11 worms for the WT and 13 worms per condition for the transgenic worms). The numbers are the irradiance values in mW mm^−2^; ret is the abbreviation for retinal. (**E**) The dependence of the pharyngeal pumping frequency on the irradiance calculated from the 30-60 s segment of the data shown in B. The symbols are the data from individual worms; the lines are the mean and SEM values. The empty and filled symbols for the transgenic worms show the data from two independently created transgenic lines. The online version of this article includes the following source data for Figure 6: Source data 1. Source data for the numerical values shown in (D, E).

To test *Ans*ACR as an optogenetic inhibitory tool in the context of an intact behaving animal, we expressed the encoding construct fused to a C-terminal EYFP tag in the *C. elegans* cholinergic neurons using the *unc-17* promoter (a scheme of the expression construct is shown in Figure 7A). To assess pharyngeal function non-invasively, we recorded EPGs from live animals sucked into a pipette (Raizen & Avery, 1994). An EPG contains transients reflecting pharyngeal muscle action potentials (Figure 7B), the frequency of which can be easily quantified. In the presence of 10 mM serotonin required to maintain regular pharyngeal pumping, its frequency in the dark was not significantly different in the transgene and wild-type worms (4.07 ± 0.05 and 4.19 ± 0.08 Hz, respectively, mean ± SEM, n = 26 transgenic and 11 wild-type worms, respectively; the p-value by the two-tailed Mann-Whitney test is 0.21), indicating that *Ans*ACR expression did not affect the pharyngeal function in the darkness. Figure 7C shows representative EPG recordings from a transgenic worm fed on bacteria supplemented with all-*trans*-retinal. The onset of 470-nm illumination caused an immediate inhibition of pumping in such transgene worms but not in the WT worms fed on the same bacteria or transgene worms in the absence of retinal (Figure 7D). The *C. elegans* genome encodes LITE-1 and GUR-3 UV/blue light receptors (unrelated to ChRs) responsible for photoinhibition of pharyngeal pumping at high light levels (Bhatla & Horvitz, 2015). However, no photoinhibition was detected in the absence of retinal in either wild-type or transgenic worms. This indicates that the irradiance used in our experiments was insufficient to stimulate these endogenous photoreceptors. The magnitude of the *Ans*ACR-mediated photoinhibition depended on the irradiance (Figure 7E). The maximal irradiance (2.1 mW mm^−2^) completely abolished the pumping for 15 s in all tested worms (n = 13). In five of 13 worms, individual action potentials were observed during the second half of the 30-s illumination period, an indication of adaptation. Two independently created transgenic lines showed the same degree of photoinhibition (Figure 7E, filled and empty symbols). The photoinhibition was fully reversible: after switching off the maximal-irradiance light, the pumping frequency returned to the pre-illumination level with τ ~9 s.

## Discussion

ChRs, also known as “*Chlamydomonas* sensory rhodopsins,” were first discovered as the photoreceptors guiding phototaxis and the photophobic response in the chlorophyte *C. reinhardtii* (Govorunova, Jung, Sineshchekov, & Spudich, 2004; Sineshchekov et al., 2002). Since then, ChRs have been identified in the genomes and transcriptomes of several other eukaryotic supergroups, including cryptophytes, haptophytes, stramenopiles, and alveolates (Govorunova et al., 2021; Govorunova et al., 2020; Govorunova et al., 2015). Furthermore, ChRs appear in the genomes of giant viruses, which likely facilitate the spread of ChR genes by horizontal transfer (Rozenberg et al., 2020; Zabelskii et al., 2020). Our identification and characterization of ChRs in ancyromonads, phylogenetically placed near the most commonly inferred root of the eukaryote tree (Brown et al., 2018), suggests that eukaryotes acquired ChR genes at the early steps of their evolution. Consistent with the role of the encoded proteins as phototaxis receptors directly proven in *C. reinhardtii*, ChR genes or transcripts occur only in protists that develop flagella at some stage of their life cycle. No exception is the diatom *O. aurita*, in which we also identified ChRs: flagella are lost in the vegetative state of this protist but are still present in its male gametes (Nanjappa, Sanges, Ferrante, & Zingone, 2017).

Prediction of biophysical properties such as ionic selectivity from protein sequences is a major unresolved problem in ChRs research. The *Nl*CCR sequence shows ~29% identity and ~49% similarity in the 7TM domain to each of the two ancyromonad ACRs and contains a neutral residue in the counterion position (Asp85 in BR), typical of all ACRs (Figure 1 – figure supplement 1, red arrow). Yet, *Nl*CCR does not conduct anions, showing instead permeability to Na^+^. In the earlier known ChRs, the presence of conserved Glu residues in TM2 and the TM2-TM3 loop, corresponding to Glu82, Glu83, Glu90, and Glu101 of *Cr*ChR2, correlates with cation selectivity (Govorunova et al., 2021). However, TM2 of *Nl*CCR contains no carboxylated residues (Figure 1 – figure supplement 1), which suggests a unique mechanism of cation selection in this channel. *Nl*CCR is the most blue-shifted among ancyromonad ChRs and generates larger photocurrents than the earlier known CCRs with a similar absorption maximum (Govorunova et al., 2013), which makes it a good candidate for optogenetic stimulation of neuronal activity.

All ancyromonad ChRs absorb light in the blue spectral range. The λ_max_ of retinylidene proteins is determined by the energy gap between the electronic ground (S0) and first excited (S1) state of the chromophore, dependent on the chromophore geometry, the protonation state of the Schiff base counterion, and the interaction of the chromophore with other residues of the retinal-binding pocket (Engqvist et al., 2015; Ernst et al., 2014; Kato et al., 2015). Paradoxically, the retinal-binding pockets of all three ancyromonad ChRs contain the residues corresponding to Met118 and Ala215 of bacteriorhodopsin, the well-known “color switches” characteristic of red-shifted microbial rhodopsins. The role of the near-ring Met118 homolog in red-shifting the spectrum has been experimentally verified in *Haloquadratum walsbyi* BR (Sudo et al., 2013), *Gloeobacter violaceus* rhodopsin (Engqvist et al., 2015), archaeorhodopsin-3 (Kato et al., 2015), and Chrimson (Oda et al., 2018). It is thought that the bulky Met side chain pushes away the C7 atom of retinal, increasing the ring-chain coplanarity, expanding the *π* conjugation, and red-shifting absorbance, as theoretically predicted in the *Gt*ACR1_C133M mutant (Tsujimura et al., 2021). The red-shifting effect of the Ala215 homolog has been demonstrated in *N. pharaonis* sensory rhodopsin II (K. Shimono et al., 2000), *H. salinarum* BR (Spudich et al., 2012), Chrimson (Oda et al., 2018), sodium-pumping rhodopsin KR2 (Inoue et al., 2019), and *Mantoniella squamata* ACR1 (Oppermann et al., 2024), and is explained by electrostatic interactions between the polar residue in this position and the RSB. However, in ancyromonad ChRs, mutations of the Met118 homolog to smaller residues and the Ala215 homolog to polar residues caused a red rather than blue spectral shift or no shift. Furthermore, the mutagenetic introduction of the ring-rotating residues responsible for the blue-shifted spectra of *Hyphochytrium catenoides* kalium channelrhodopsin 2 (Tajima et al., 2023) and *Kn*ChR (Wang et al., 2024) did not change the ancyromonad ChR spectra, which suggests that either the torsion around the C6-C7 bond is already enforced by a different geometry of the Met118 homolog or that their blue-shifted absorbance arises by a different mechanism. Atomic structures of ancyromonad ChRs are needed to investigate their paradoxical retinal binding pockets and the reasons for their unusual spectral properties.

ACRs are widely used to inhibit neuronal activity with light. We evaluated *Ans*ACR and *Ft*ACR as neuronal silencers in mouse brain slices and *Ans*ACR in the context of a live animal, the nematode *C. elegans*. We previously showed that *Gt*ACRs could inhibit action potentials at the soma while triggering synaptic transmission due to high axonal Cl^−^ reversal potential (Messier et al., 2018). *Ans*ACR and *Ft*ACR showed similar phenomena in our brain slice experiments. These ACRs can inhibit action potentials in cortical neurons but depolarize axonal terminals and trigger synaptic transmission. Fusing these new ACRs with somatodendritic trafficking motifs to reduce axonal expression (Mahn et al., 2018; Messier et al., 2018) could lead to potent inhibition with reduced axonal excitation.

Optogenetic inhibition of *C. elegans* pharyngeal pumping has been demonstrated earlier upon expression of the *Leptosphaeria maculans* proton-pumping rhodopsin known as Mac (Trojanowski, Padovan-Merhar, Raizen, & Fang-Yen, 2014) or *N. pharaonis* halorhodopsin (*Np*HR) (Schüler, Fischer, Shaltiel, Steuer Costa, & Gottschalk, 2015) in the cholinergic neurons. However, ion-pumping rhodopsins such as these transport only one ion per absorbed photon and, therefore, require almost 20 times higher irradiance for photoinhibition than the maximal irradiance used in this study. All ACRs, including *Ans*ACR that we tested in the worms, transport multiple anions during the open state and, therefore, are more efficient optogenetic silencers than the ion-pumping rhodopsins. The partial recovery of pharyngeal pumping that we observed after 15-s illumination, even at the highest tested irradiance, most likely reflects an adaptation of the pharyngeal muscles capable of generating autonomous contractions in the presence of acetylcholine tonically released from the pharyngeal neurons (Trojanowski et al., 2016).

*Ans*ACR exhibited robust 2P excitation, with action spectra showing a maximum at ~920 nm. This wavelength aligns well with the excitation range of common Ti:Sapphire lasers, widely used in neuroscience laboratories. These findings show that *Ans*ACR can be deployed for *in vivo* optogenetic silencing with single-cell or subcellular precision, expanding the toolkit for precise manipulations in neuroscience research. Our characterization of ancyromonad ChRs has contributed to a better understanding of light-gated channel function and yielded superior blue-shifted tools for optogenetic excitation and inhibition.

## Materials and Methods

### Bioinformatics and molecular biology

The ChR homologs from *Ancyromonas sigmoides* strain B-70 (CCAP1958/3), *Fabomonas tropica* strain NYK3C, *Nutomonas longa* strain CCAP 1958/5 (Brown et al., 2018; Torruella et al., 2015), and *Ancoracysta twista* strain TD-1 (Janouškovec et al., 2017) were identified in the EukProt V3 database (Richter et al., 2022) using Sequnceserver BLASTP (Priyam et al., 2019). The *A. sigmoides*, *F. tropica*, and *A. twista* ChR sequences are available from Dr. Andrey Rozenberg's ChR database (Rozenberg, 2024). The metagenomic homolog 1 was found by Sequnceserver BLASTP in the TARAeuCatV2 database (Sunagawa et al., 2015) accessed at the KAUST Metagenomic Analysis Platform (KMAP) (Alam et al., 2021). The metagenomic homolog 2 was found using the search mode of BLASTP in the MATOU database (Marine Atlas of Tara Oceans Unigene plus metaG eukaryotes) (Villar et al., 2018) with the query sequence of *Nl*CCR. The *Odontella aurita* strain CCMP816 homologs GHBW01284417 and GHBW01118808 were identified by TBLASTN in the National Center of Biological Information (NCBI) transcriptome shotgun assembly (TSA) project GHBW00000000. The *Paraphysoderma* homolog (*Pars*R) was found in the *P. sedebokerense* strain JEL821 v. 1.0 genome assembly (Amses et al., 2022) by the annotation text search using bacteriorhodopsin as a keyword at the Mycocosm portal (Grigoriev et al., 2014).

The protein alignment was created using the MUSCLE algorithm with default parameters implemented in MegAlign Pro software v. 17.1.1 (DNASTAR Lasergene, Madison, WI) and truncated after the end of TM7. Phylogeny was analyzed with IQ-TREE v. 2.1.2 (Minh et al., 2020) using automatic model selection and ultrafast bootstrap approximation (1,000 replicates) (Hoang, Chernomor, von Haeseler, Minh, & Vinh, 2018). The best tree was visualized and annotated using iTOL v. 7 (Letunic & Bork, 2024).

For expression in human embryonic kidney (HEK293) cells, mammalian codon-optimized polynucleotides encoding amino acid residues 1-265 of the *A. sigmoides* homolog, 1-268 of the *F. tropica* homolog, 1-272 of the *N. longa* coding homolog, 1-243 of the *A. twista* homolog, 1-241 of the GHBW01284417 *O. aurita* homolog, 1-242 of the GHBW01118808 *O. aurita* homolog, and 1-336 of the *P. sedebokerense* homolog were synthesized, fused to a C-terminal mCherry tag, and cloned into the pcDNA3.1(+) vector (Invitrogen, Cat. #V19520) at GenScript. For expression in *Pichia*, the constructs were fused with the C-terminal 8His-tag and cloned in the pPICZalpha-A vector (Invitrogen, Cat. #V19520). A QuikChange XL site-directed mutagenesis kit (Agilent Technologies, Cat. #200516) was used to introduce point mutations. For expression in the mouse cortical neurons, *Ans*ACR and *Ft*ACR were tagged with EYFP (enhanced yellow fluorescent protein) at the C-terminus and cloned into the pAAV-CAG vector.

### HEK293 cell culture and transfection

No cell lines from the list of known misidentified cell lines maintained by the International Cell Line Authentication Committee were used in this study. HEK293 cells used in 1P excitation experiments were obtained from the American Type Culture Collection (ATCC; Cat. #CRL-1573). The cells were plated on 2-cm diameter plastic dishes 48-72 hrs before experiments, grown for 24 hrs, and transfected with 10 μl of Lipofectamine LTX with Plus Reagent (ThermoFisher, Cat. #15338100) using 3 μg DNA per dish for manual patch clamping, and 6 μg DNA per dish for automated patch clamping. All-*trans*-retinal (Millipore-Sigma, Cat. #116-31-4) was added immediately after transfection at the final concentration of 5 μM. For 2P excitation experiments, HEK293A cells were plated on 30-70 kDa poly-*d*-lysine-coated 12-mm circular coverslips (Carolina cover glass #0, Cat. #633009) in 24-well plates (P24-1.5H-N, Cellvis) at 30% confluence, transfected with 1.2 μL FuGENE HD transfection reagent (Promega, Cat. #E2311) using 200 ng DNA per well 48-72 h before measurements, and supplemented with all-*trans*-retinal as described above.

### Automated whole-cell patch clamp recording from HEK293 cells

Automated patch clamp recording was conducted at room temperature (21°C) 48-72 h after transfection with a SyncroPatch 384 (Nanion Technologies) based on a Biomek i5 automated liquid handler (Beckman Coulter), using NPC-384T S-type chips (Nanion, Cat. #222101) with one hole per well, as described earlier (Govorunova, Sineshchekov, Brown, & Spudich, 2022). Before measurements, transfected cells were dissociated using TrypLE Express, diluted with CHO-S-SFM-II medium (both from ThermoFisher, Cat.# 12604013 and 31033020, respectively) and resuspended in External Physiological solution (Nation, Cat.# 08 3001). The compositions of this and other solutions used in automated patch clamp recordings and the corresponding liquid junction potential (LJP) values calculated using the ClampEx LJP calculator are listed in Supplementary Table 1. The voltages in all IV curves for HEK293 cells in this manuscript were corrected for LJPs; the holding voltage values in the figures showing traces correspond to the amplifier output before the LJP subtraction. Illumination was provided with LUXEON Z Color Line light-emitting diodes (LEDs) Cat.# LXZ1-PB01 (470 ± 10 nm) arranged in a 6×16 matrix. The forward LED current was 900 mA, the illumination duration was 200 ms (limited by the LED duty cycle), and the interval between successive light pulses was 60 s. The LEDs were driven by a derivative of CardioExcyte 96 SOL (Nanion, Cat. #191003) and controlled by Biomek commands. PatchControl384 v. 2.3.0 (Nanion Technologies) software was used for data acquisition at a 5 kHz sampling rate (200 μs per point). The photocurrent amplitudes at the peak and the end of illumination were calculated using DataControl384 software v. 2.3.0 (Nanion Technologies). Further analysis was performed using the Origin Pro 2016 software (OriginLab Corporation).

### Manual patch clamp recording using 1P excitation in HEK293 cells

Manual patch clamp recordings were performed with an Axopatch 200B amplifier (Molecular Devices). The pipette solution contained (in mM) KCl 130, MgCl_2_ 2, HEPES 10 pH 7.4, and the bath solution contained (in mM) NaCl 130, CaCl_2_ 2, MgCl_2_ 2, glucose 10, HEPES 10 pH 7.4. In experiments to test the relative permeability of ChRs for Cl^−^, NaCl in the bath was replaced with Na aspartate. The low-pass filter of the amplifier output was set to 2 kHz. The signals were digitized with a Digidata 1440A (Molecular Devices) at a 5 kHz sampling rate (200 μs per point) using pClamp 10.7. Patch pipettes with 2-3 MΩ resistances were fabricated from borosilicate glass. Laser excitation was provided by a Minilite Nd:YAG laser (532 nm, pulse width 6 ns, energy 5 mJ; Continuum). The current traces were logarithmically filtered using Logpro software (Spudich, 2022). Curve fitting was performed using Origin Pro software. Continuous light pulses were provided by a Polychrome V light source (T.I.L.L. Photonics GMBH) in combination with a mechanical shutter (Uniblitz Model LS6, Vincent Associates; half-opening time 0.5 ms). The action spectra of photocurrents were constructed by calculating the initial slope of photocurrent recorded in response to 15-ms light pulses at the intensity <25 μW mm^−2^, corrected for the quantum density measured at each wavelength, and normalized to the maximal value.

### Manual patch clamp recording using 2P excitation in HEK293A cells

HEK293A cells were transfected using the pAAV-CAG-*Ans*ACR-EYFP plasmid. 2P excitation of *Ans*ACR was conducted on an inverted microscope with multiphoton capability (A1R-MP, Nikon Instruments) at room temperature. A coverslip seeded with the transfected cells was placed in a custom glass-bottom chamber based on Chamlide EC (Live Cell Instrument) with a glass bottom made with a 24×24 mm cover glass #1 (Erie Scientific, Cat. #89082-270). Cells were perfused continuously with the external solution described in the 1P excitation section. Whole-cell voltage-clamp recordings were performed using a MultiClamp 700B amplifier (Molecular Devices). The cells were held at −20 mV, with the command voltage compensated for the 4.4 mV LJP calculated using the ClampEx v.11.1 (Molecular Devices) built-in calculator. The signal was digitized with an Axon Digidata 1550B1 Low Noise system with HumSilencer (Molecular Devices), and the current was recorded at 10 kHz using pClamp.

The near-IR excitation was generated by a titanium:sapphire femtosecond laser (Chameleon Ultra II, Coherent) with a repetition rate of 80 MHz and a tuning range between 680 and 1,080 nm. Laser pulses were not pre-compensated for dispersion in the microscope optical path. Laser power was tuned using an acousto-optic modulator and delivered to the sample plane through a 40x0.95-numerical aperture (NA) objective (CFI Plan Apochromat Lambda, Nikon Instruments). Scanning across xy regions-of-interest was achieved using a resonant scanning microscope (Nikon A1RMP) at 33.3 Hz. An illumination “pulse” consisted of 30 sequential raster scans (total duration ~1 s) over a 40.96×40.96 μm (512×512 pixels) area, selected to approximate the average size of a HEK293A cell, and no time gap between each scan. To determine the 2P action spectra of *Ans*ACR, the excitation wavelength was varied from 800 to 1,080 nm in 40-nm increments. The laser was tuned to 7.5 mW at the sample plane at each wavelength measured at the sample plane with a microscope slide power sensor (S170C, Thorlabs). This power level was chosen to obtain robust photocurrents while remaining at or near the quadratic regime, where the slope of initial photocurrents quadruples with a doubling of the excitation power. Deviations from the target power level of 7.5 mW (<10%) were corrected by considering the quadratic dependence of photocurrents on power under 2P excitation. To mitigate desensitization, we spaced illumination pulses ~55 s apart. We verified that the power ramp and spectral scan protocols caused <20% reduction of the peak photocurrent, measured during 7.5-mW, 920-nm light pulses applied at the start and end of each protocol. The 2P action spectrum was constructed by measuring the initial linear slope of the photocurrent rise at each wavelength and plotted using Origin Pro 2016 software (OriginLab Corporation).

### Expression and purification of ancyromonad ACRs from Pichia pastoris

The plasmids carrying the expression constructs were linearized with Sac I and delivered into the *P. pastoris* strain SMD1168 by electroporation. A single colony resistant to 0.25 mg/ml zeocin was picked and inoculated into buffered complex glycerol medium, after which the cells were transferred to buffered complex methanol (0.5%) medium supplemented with 5 μM all-*trans*-retinal (Millipore-Sigma, Cat. # 116-31-4) and grown at 30 °C with shaking at 230 rpm. After 24 h, the yellow-colored cells were harvested by centrifugation at 5000 g for 10 min, and the cell pellets were resuspended in 100 ml ice-cold buffer A (20 mM HEPES, pH 7.4, 150 mM NaCl, 1 mM EDTA, 5% glycerol) and lysed by either French press or bead beater. After centrifugation at low speed (5000 g for 10 min) to remove cell debris, membrane fractions were pelleted at 190,000 g for 1 h using a Ti45 Beckman rotor. The membranes were suspended in Buffer B (350 mM NaCl, 5% glycerol, 20 mM HEPES, pH 7.5) with 1 mM phenylmethylsulfonyl fluoride and solubilized with 1% n-dodecyl-β-D-maltoside (DDM; Anatrace, Cat. # D310) for 1 h at 4 °C with shaking. Undissolved content was removed after ultracentrifugation using a Ti45 rotor at 110,000 g for 1 h. The supernatant supplemented with 15 mM imidazole was incubated with nickel-nitrilotriacetic acid resin (Qiagen, Cat. # 30210) for 1 h with shaking at 4 °C. The resin was washed step-wise using 15 mM and 40 mM imidazole in Buffer B supplemented with 0.03% DDM. The protein was eluted with 400 mM imidazole and 0.03% DDM in buffer B. Protein fractions were pooled and concentrated using a 50 kDa MWCO Amicon Ultra Centrifugal Filter (Millipore-Sigma, Cat. # UFC9050), flash-frozen in liquid nitrogen and stored at −80 °C until use.

### Absorption spectroscopy and flash photolysis

Absorption spectra of detergent-purified protein samples were recorded using a Cary 4000 spectrophotometer (Varian). Photoinduced absorption changes were measured with a laboratory-constructed crossbeam apparatus. Excitation flashes were provided by a Minilite II Nd:YAG laser (532 nm, pulse width 6 ns, energy 5 mJ; Continuum). Measuring light was from a 250-W incandescent tungsten lamp and a McPherson monochromator (model 272, Acton). Absorption changes were detected with a Hamamatsu Photonics photomultiplier tube (model R928) combined with a second monochromator of the same type. Signals were amplified by a low noise current amplifier (model SR445A; Stanford Research Systems) and digitized with a GaGe Octopus digitizer board (model CS8327, DynamicSignals LLC), with a maximal sampling rate of 50 MHz. Logarithmic data filtration was performed using the GageCon program (Sineshchekov, Govorunova, Li, Wang, & Spudich, 2023).

### Mice

All procedures to maintain and use mice were approved by the Institutional Animal Care and Use Committee at Baylor College of Medicine (protocol AN-6544). Mice were maintained on a 14 hr:10 hr light:dark cycle with regular mouse chow and water ad libitum. The temperature was maintained at 21–25°C and humidity at 40-60%. Experiments were performed during the light cycle. Female ICR (CD-1) mice were purchased from Baylor College of Medicine Center for Comparative Medicine, and male C57BL6/J (JAX #000664) mice were obtained from Jackson Laboratory. Both male and female mice were used in the experiments.

### In utero electroporation

Female ICR mice were crossed with male C57BL6/J mice to obtain timed pregnancies. *In utero* electroporation was used to deliver the transgenes (Xue, Atallah, & Scanziani, 2014). To express *Ans*ACR or *Ft*ACR in the layer 2/3 pyramidal neurons of the somatosensory cortex, pAAV-CAG-*Ans*ACR-EYFP or pAAV-CAG-*Ft*ACR-EYFP (2.5 μg μl^−1^ final concentration) was mixed with pCAG-tdTomato (0.1 μg μl^−1^ final concentration) and Fast Green (Sigma-Aldrich, 0.01% final concentration) for injection. On embryonic day 15, pregnant mice were anesthetized, and a beveled glass micropipette (tip size 100-μm outer diameter, 50-μm inner diameter) was used to penetrate the uterus and the embryo skull to inject ~1.5 μl DNA solution into one lateral ventricle. Five pulses of current (voltage 39 V, duration 50 ms) were delivered at 1 Hz with a Tweezertrode (5-mm diameter) and a square-wave pulse generator (Gemini X2, BTX Harvard Bioscience). The electrode paddles were positioned in parallel with the brain’s sagittal plane. The cathode contacted the side of the brain ipsilateral to the injected ventricle to target the somatosensory cortex. Transfected pups were identified by the transcranial fluorescence of tdTomato with an MZ10F stereomicroscope (Leica) 1 day after birth.

### Brain slice electrophysiology and imaging

Mice were used at the age of 4-6 weeks for acute brain slice electrophysiology experiments. Mice were anesthetized by an intraperitoneal injection of a ketamine and xylazine mix (80 mg kg^−1^ and 16 mg kg^−1^, respectively) and perfused transcardially with cold (0-4°C) slice cutting solution containing 80 mM NaCl, 2.5 mM KCl, 1.3 mM NaH_2_PO_4_, 26 mM NaHCO_3_, 4 mM MgCl_2_, 0.5 mM CaCl_2_, 20 mM *d*-glucose, 75 mM sucrose and 0.5 mM sodium ascorbate (315 mOsm l^−1^, pH 7.4, saturated with 95% O_2_/5% CO_2_). Brains were removed and sectioned in the cutting solution with a VT1200S vibratome (Leica) to obtain 300 μm coronal slices. Slices were incubated in a custom-made interface holding chamber containing slice cutting solution saturated with 95% O_2_/5% CO_2_ at 34°C for 30 min and then at room temperature for 20 min to 10 h until they were transferred to the recording chamber. We performed recordings on submerged slices in artificial cerebrospinal fluid (ACSF) containing 119 mM NaCl, 2.5 mM KCl, 1.3 mM NaH_2_PO_4_, 26 mM NaHCO_3_, 1.3 mM MgCl_2_, 2.5 mM CaCl_2_, 20 mM *d*-glucose and 0.5 mM sodium ascorbate (305 mOsm l^−1^, pH 7.4, saturated with 95% O_2_/5% CO_2_, perfused at 3 ml min^−1^) at 30-32°C. For whole-cell recordings, a K^+^-based pipette solution containing 142 mM K^+^ gluconate, 10 mM HEPES, 1 mM EGTA, 2.5 mM MgCl_2_, 4 mM ATP-Mg, 0.3 mM GTP-Na, 10 mM Na_2_-phosphocreatine (295 mOsm l^−1^, pH 7.35) was used. Membrane potentials reported in Figure 6 and Figure 6 – figure supplement 1 were not corrected for LJP, which was 12.5 mV as measured experimentally. Neurons were visualized with video-assisted IR differential interference contrast imaging, and fluorescent neurons were identified by epifluorescence imaging under a water immersion objective (×40, 0.8 NA) on an upright SliceScope Pro 1000 microscope (Scientifica) with an IR-1000 CCD camera (DAGE-MTI). Data were acquired at 10 kHz and low-pass filtered at 4 kHz with an Axon Multiclamp 700B amplifier and an Axon Digidata 1440 A Data Acquisition System under the control of Clampex 10.7 (Molecular Devices). Data were analyzed offline using Clampfit (Molecular Devices). For photostimulation, blue light was emitted from a collimated 470 nm light-emitting diode (LED; M470L3, Thorlabs) to stimulate *Ans*ACR- or *Ft*ACR-expressing neurons. The LEDs were driven by a LED driver (Throlabs LEDD1B) under the control of an Axon Digidata 1440 A Data Acquisition System and Clampex 10.7. The light was delivered through the reflected light fluorescence illuminator port and the ×40 objective.

To evaluate inhibition efficiency, action potentials of *Ans*ACR- or *Ft*ACR-expressing neurons were evoked by injecting a series of 1.5-s depolarizing current pulses (−0.1-0.5 nA) in whole-cell current clamp mode. 1-s 470 nm (38.7 mW mm^−2^) light stimulation was applied in the middle of current injections with 30-s inter-trial-interval. Resting membrane potentials, input resistances, and capacitances were measured in the trials with −0.1 nA current injection. To examine the excitatory effect of the ACRs, 10-ms 470 nm (38.7 mW mm^−2^) light stimulation was applied, and ACR^−^ neurons were clamped at −70 mV to record excitatory post-synaptic currents. Only recordings with R_a_ < 20 MΩ were included in the analysis.

After electrophysiology recordings, fluorescent images of the brain slices were acquired on an Axio Zoom.V16 Fluorescence Stereo Zoom Microscope (Zeiss) and processed using MATLAB2024b (MathWorks). Images were taken from 20 brain slices of two male and two female mice.

### Generation of transgenic C. elegans strains and EPG recording

The transgenic *C. elegans* strains COP2831 and COP2832 [*uncp*-17::*Ans*ACR::EYFP::*tbb-2u*], *unc-119*(+))} II; *unc-119(ed3)* III expressing *Ans*ACR in cholinergic neurons were created by InVivo Biosystems using the Mos1-mediated Single Copy Insertion (MosSCI) method, which enables integrating the transgene as a single-copy insertion at a designated locus in the *C. elegans* genome (Frokjaer-Jensen, 2015). *Unc*-119 rescue cassette insertion was used to bring the transgene into a Mos1 target locus on chromosome II and create rescue of the function of the *unc-119(ed3)* III mutant allele. The Mos1 locus was selected for position-neutral effects and to avoid the gene coding regions, introns, and transcription factor binding sites. The integration of the transgene was confirmed by PCR.

The transgenic and Bristol N2 wild-type worms were grown at 20°C on *E. coli* strain OP50 lawns in the absence or presence of 10 μM (final concentration) all-*trans*-retinal (Millipore-Sigma, Cat. # 116-31-4), which was mixed with the bacteria before seeding Nematode Growth Medium (NGM) plates. EPG recordings were performed from intact worms sucked into a pipette (Raizen & Avery, 1994). The pipettes (200 kΩ resistance) were pulled from borosilicate glass and filled with the External Physiological solution, the composition of which is specified in the above section. The worms were transferred to the same solution supplemented with 10 mM serotonin before the measurements. The data were acquired in the voltage clamp mode of the same Axopatch 200B amplifier used for manual patch clamp recording from HEK293 cells and the same software. The data obtained in two independently created strains were pooled together. The photoexcitation was provided by the Polychrome V light source described above. The frequency of the R1-spikes was calculated using the Event Detection by the Threshold Search function of ClampFit after applying a 2-Hz high-pass digital filter. Further analysis was performed using the Origin Pro 2016 software.

### Reproducibility and statistics

Plasmids encoding different ChR variants were randomly assigned to transfect identical cell batches. Three independent transfections were performed on different experimental days; the data obtained were pooled together. In automated patch clamp studies, cells were randomly drawn into the wells. For an unbiased estimation of the photocurrent amplitude, the data from wells that formed seals with a resistance <500 MΩ were excluded. For a more accurate estimation of the V_r_ values by plotting the IV curves, wells with a seal resistance <500 MΩ and photocurrents of the absolute magnitude <50 pA at −80 mV were excluded. In manual patch clamp experiments, the cells were selected for patching by inspecting their tag fluorescence; non-fluorescent cells and cells in which no GΩ seal was established or lost during recording were excluded from the analysis. In automated and manual patch clamp experiments, the photocurrent traces recorded from different cells transfected with the same construct were considered biological replicates (reported as n values). These values indicate how often the experiments were performed independently.

Statistical analysis of the patch clamp data was performed using Origin Pro 2016 software. The normal distribution of the data was not assumed. The non-parametric two-tailed Mann-Whitney and Kolmogorov-Smirnov tests were used to compare the means. No statistical methods were used to pre-determine sample sizes, but the sample sizes were similar to those reported in the previous publications (Govorunova, Gou, et al., 2022; Morizumi et al., 2023). }. Statistical analysis of the cortical neuron patch clamp data was performed using ClampFit 10.7 (Molecular Devices) and Prism 10.3 (GraphPad).

## Supplementary Material

Supplement 1

## Data Availability

The numerical data and statistical analyses are provided in the Suppl. Data File 1. The sequence information was deposited at the NCBI with GenBank accession numbers PQ657777-PQ657783. The plasmids encoding *Ans*ACR, *Ft*ACR, and *Nl*CCR expression constructs in the pcDNA3.1 vector backbone were deposited at Addgene (plasmids #232598, 232599, and 232600, respectively). The plasmids pAAV-CAG-*Ans*ACR-EYFP and pAAV-CAG-*Ft*ACR-EYFP were deposited at Addgene (plasmids #XXX and #YYY, respectively).
