# Supplementary material for: Blue-shifted ancyromonad channelrhodopsins for multiplex optogenetics
Source: bioRxiv. 2025 Feb 27:2025.02.24.639930. Preprint. [Version 1] doi: 10.1101/2025.02.24.639930 (PMC11888301; doi:10.1101/2025.02.24.639930)
Supplement: Supplement 1 [file NIHPP2025.02.24.639930v1-supplement-1.pdf]

bioRxiv preprint doi: https://doi.org/10.1101/2025.02.24.639930; this version posted March 1, 2025. The copyright holder for this preprint (which was not certified by peer review) is the author/funder, who has granted bioRxiv a license to display the preprint in perpetuity. It is made available under aCC-BY 4.0 International license.

Figure supplement and their legends

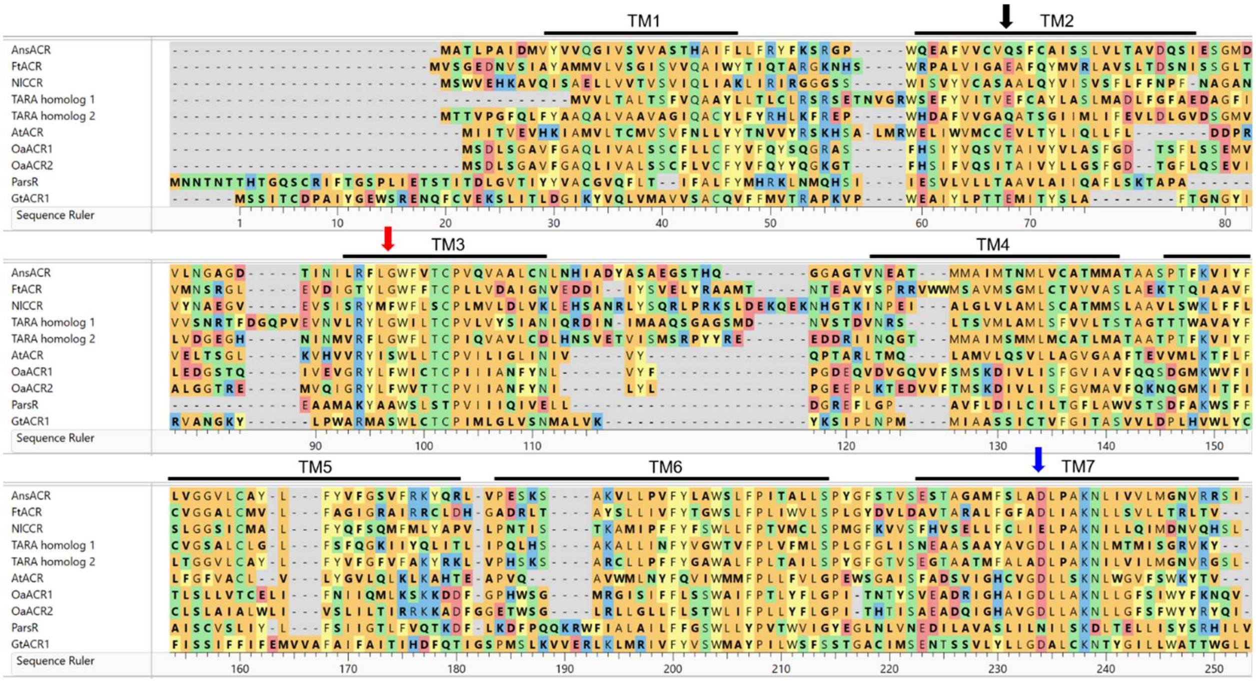

Figure 1 – figure supplement 1. The protein alignment of the 7TM domains of ChR variants identified and characterized in this study and the previously known *Gt*ACR1.

The amino acid residues are colored according to their chemical properties. The residue ruler is according to the *Gt*ACR1 sequence. The lines show the transmembrane helices TM1-TM7 of *Gt*ACR1. The arrows point to the positions corresponding to Asp68 (black), Ser97 (red), and Asp234 (blue) of *Gt*ACR1.

bioRxiv preprint doi: https://doi.org/10.1101/2025.02.24.639930; this version posted March 1, 2025. The copyright holder for this preprint (which was not certified by peer review) is the author/funder, who has granted bioRxiv a license to display the preprint in perpetuity. It is made available under aCC-BY 4.0 International license.

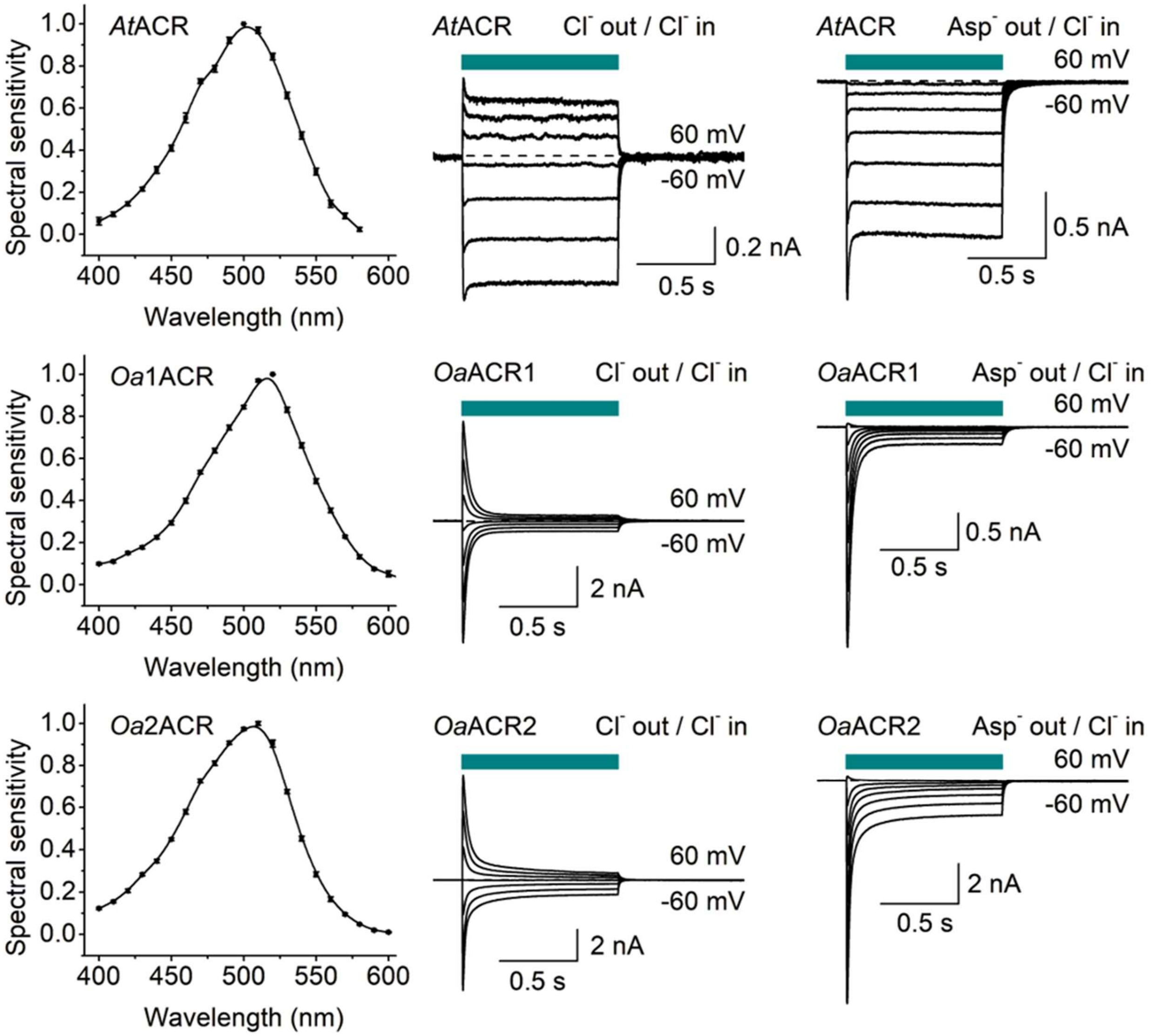

Figure 1 – figure supplement 2. Action spectra and photocurrents of ACRs from *Ancoracysta twista* and *Odontella aurita.*

In the left panels, the action spectra. The symbols are the mean values, and the error bars are SEM values; n = 6 cells for each variant. In the middle and right panels, the photocurrents recorded by manual patch clamping in the Cl^- bath (middle) and Asp^- bath (right) at the holding voltages increased in 20-mV increments from -60 mV. The dark cyan bars show the duration of illumination (500, 520 and 510 nm, respectively, for *At*ACR, *Oa*ACR1, and *Oa*ACR2).

bioRxiv preprint doi: https://doi.org/10.1101/2025.02.24.639930; this version posted March 1, 2025. The copyright holder for this preprint (which was not certified by peer review) is the author/funder, who has granted bioRxiv a license to display the preprint in perpetuity. It is made available under aCC-BY 4.0 International license.

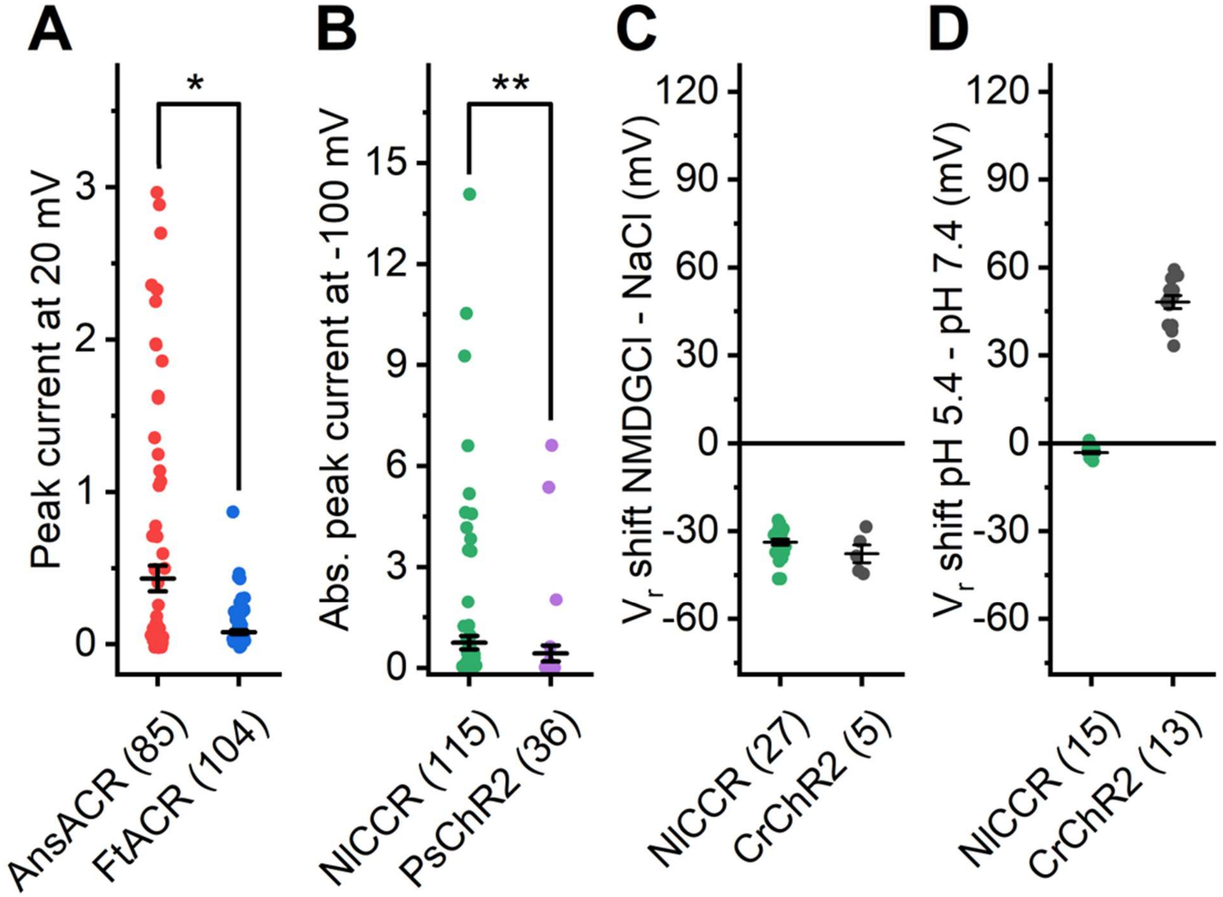

Figure 2 – figure supplement 1. Photocurrent amplitudes of anycromonad ChRs and comparison of *Nl*CCR with *Cr*ChR2.

(**A, B**) Unbiased estimation of peak photocurrent amplitudes. The photocurrents were recorded at 20 mV for *Ans*ACR and *Ft*ACR, and at -60 mV for *Nl*CCR and *Ps*ChR2. *, p = 0.0047; **, p = 9.9E10-6 by the two-tailed, two-sample Kolmogorov-Smirnov test. (**C, D**) The V_r shifts measured upon replacing Na^+ with $NMDG^+$ in the external solution (C), and upon its acidification from pH 7.4 to 5.4 (D). In all panels, the symbols are the data from individual cells; the lines are the mean and SEM values; the numbers in brackets are the numbers of cells sampled.

The online version of this article includes the following source data for Figure 2 – figure supplement 1:
Source data 1. Source data for the numbers of cells sampled and numerical values shown in (A-D).

bioRxiv preprint doi: https://doi.org/10.1101/2025.02.24.639930; this version posted March 1, 2025. The copyright holder for this preprint (which was not certified by peer review) is the author/funder, who has granted bioRxiv a license to display the preprint in perpetuity. It is made available under aCC-BY 4.0 International license.

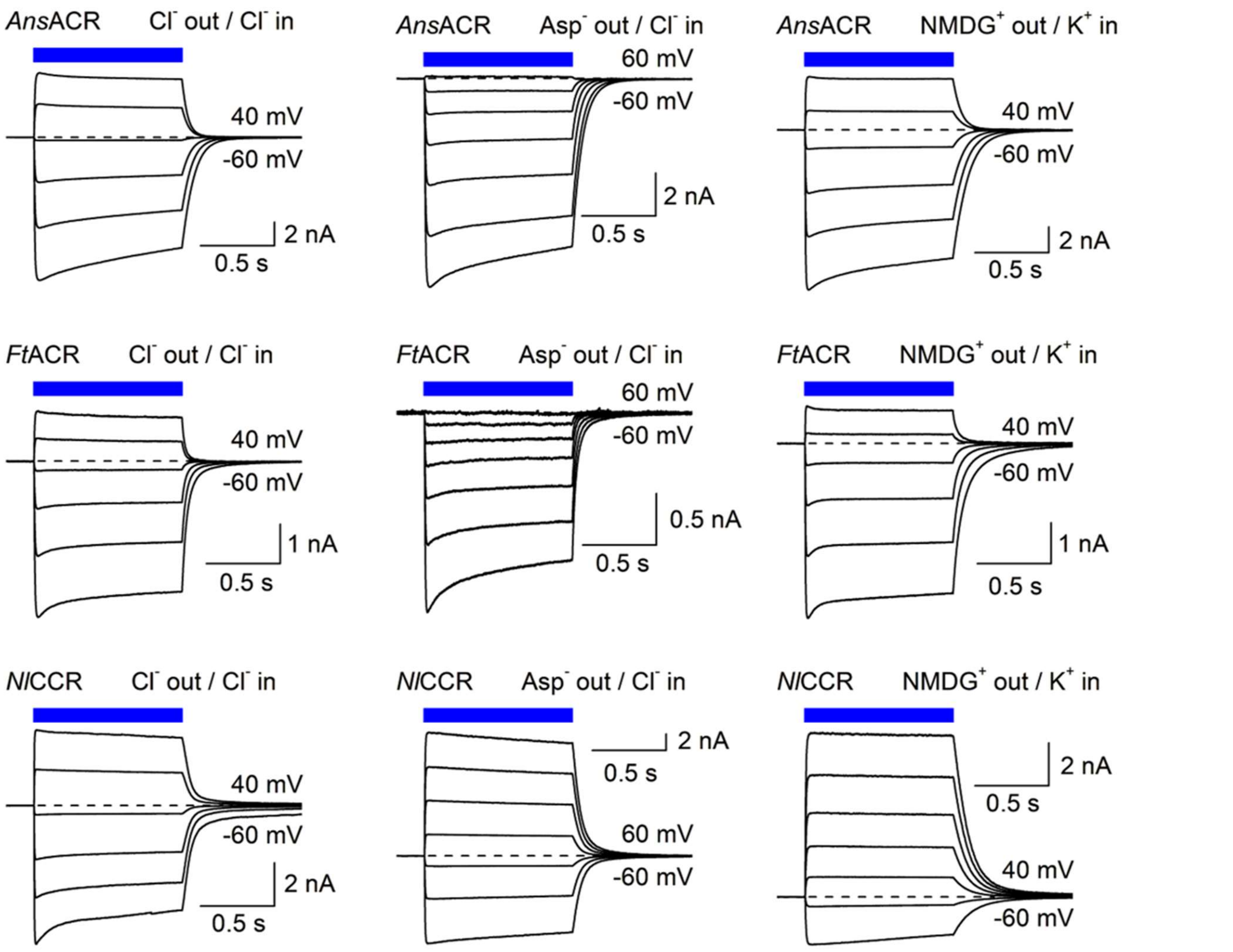

Figure 2 – figure supplement 2. Photocurrent traces recorded from ancyromonad ChRs by manual patch clamping.

The photocurrents were recorded by manual patch clamping in the Cl^- bath (left), Asp^- bath (middle), and $NMDG^+$ bath (right) at the holding voltages increased in 20-mV increments from -60 mV. The blue bars show the duration of 470 nm illumination.

bioRxiv preprint doi: https://doi.org/10.1101/2025.02.24.639930; this version posted March 1, 2025. The copyright holder for this preprint (which was not certified by peer review) is the author/funder, who has granted bioRxiv a license to display the preprint in perpetuity. It is made available under aCC-BY 4.0 International license.

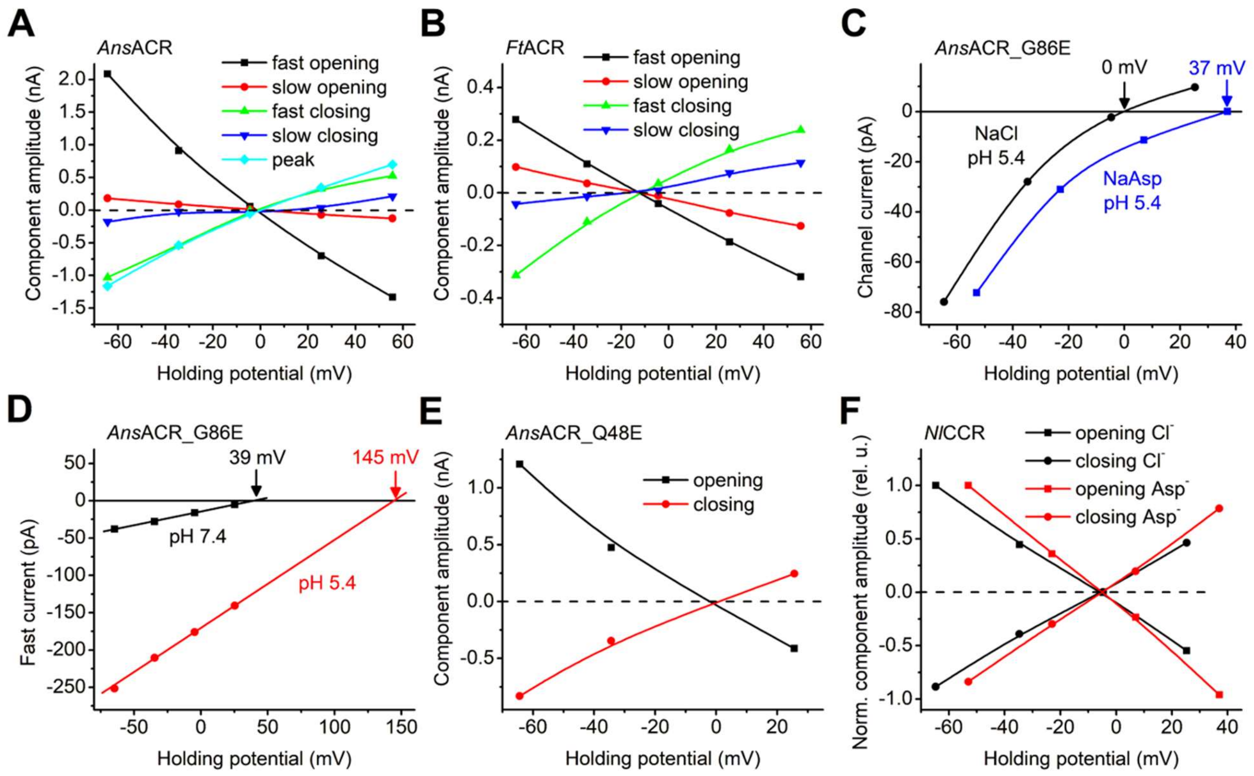

Figure 3 – figure supplement 1. Current-voltage relationships of photocurrent kinetic components. The amplitudes of the channel closing and opening components were estimated by multi-componential approximation of the laser-flash-evoked photocurrents.

The online version of this article includes the following source data for Figure 3 – figure supplement 1: Source data 1. Source data for the numerical values shown in (A-F).

bioRxiv preprint doi: https://doi.org/10.1101/2025.02.24.639930; this version posted March 1, 2025. The copyright holder for this preprint (which was not certified by peer review) is the author/funder, who has granted bioRxiv a license to display the preprint in perpetuity. It is made available under aCC-BY 4.0 International license.

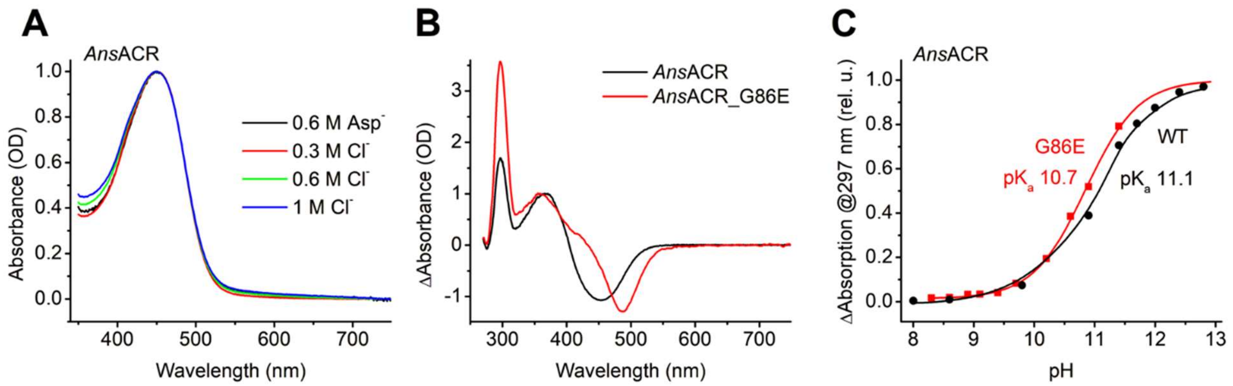

Figure 4 – figure supplement 1. Probing the RSB region in the wild-type *Ans*ACR and *Ans*ACR_G86E mutant.
(**A**) The absorption spectra of detergent-purified *Ans*ACR at the indicated Cl^- concentrations. (**B**) The difference spectra obtained upon alkalization. (**C**) pH titration of the absorbance difference at 297 nm.

The online version of this article includes the following source data for Figure 4 – figure supplement 1:
Source data 1. Source data for the numerical values shown in (C).

bioRxiv preprint doi: https://doi.org/10.1101/2025.02.24.639930; this version posted March 1, 2025. The copyright holder for this preprint (which was not certified by peer review) is the author/funder, who has granted bioRxiv a license to display the preprint in perpetuity. It is made available under aCC-BY 4.0 International license.

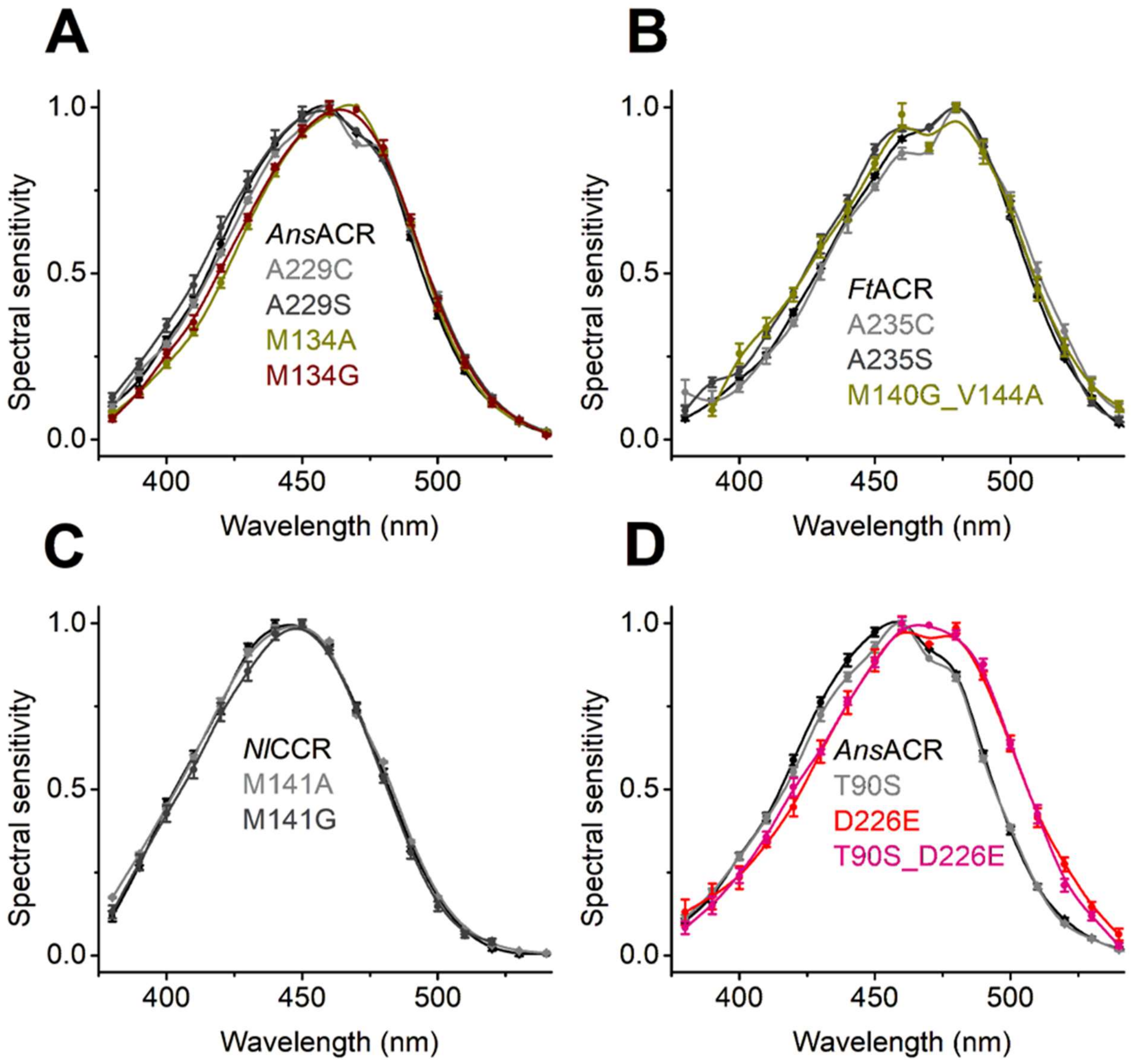

963 **Figure 5 – figure supplement 1. Mutations blue-shifting other microbial rhodopsins spectra do not affect**
964 **ancyromonad ChRs.**
965 The photocurrent action spectra of the indicated mutants compared to the respective WTs. The symbols
966 are the mean values, the error bars are SEM values.

967 The online version of this article includes the following source data for Figure 5 – figure supplement 1:
968 Source data 1. Source data for the numbers of cells sampled and numerical values shown in (A-D).

969

bioRxiv preprint doi: https://doi.org/10.1101/2025.02.24.639930; this version posted March 1, 2025. The copyright holder for this preprint (which was not certified by peer review) is the author/funder, who has granted bioRxiv a license to display the preprint in perpetuity. It is made available under aCC-BY 4.0 International license.

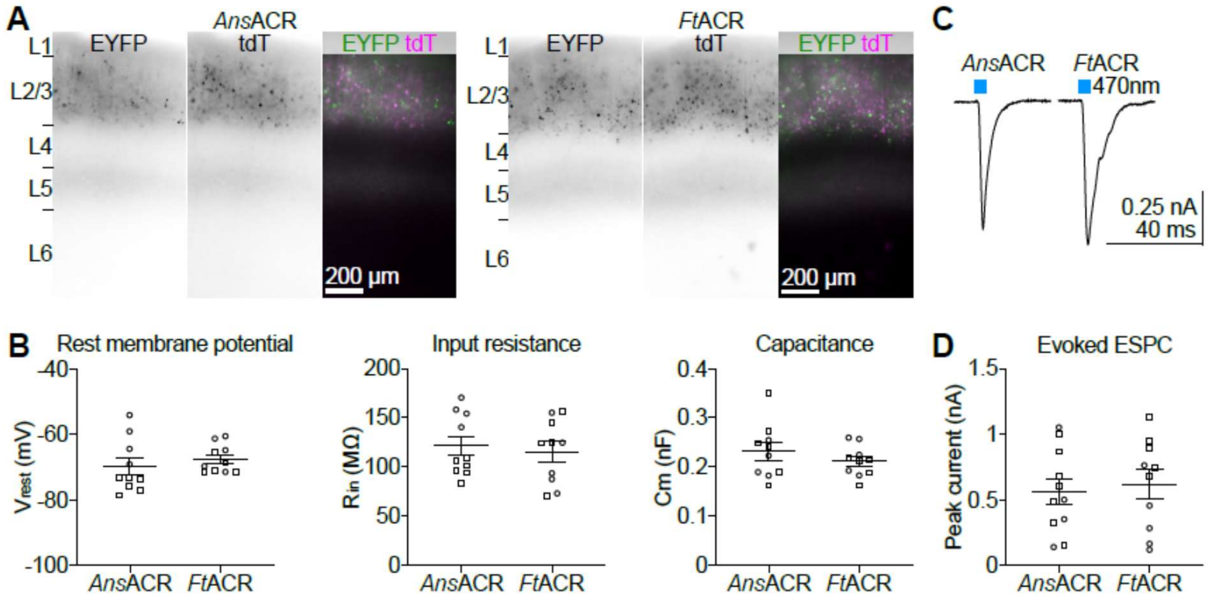

Figure 6 – figure supplement 1. Characterization of *Ans*ACR and *Ft*ACR expression in cortical neurons and axonal excitatory effect.

(**A**) Reprehensive fluorescence images of 300 µm-thick brain slices expressing tdTomato and EYFP fused to the C terminals of *Ans*ACR (left) and *Ft*ACR (right) in cortical layer 2/3 pyramidal neurons. The axons of ACR^+ layer 2/3 pyramidal neurons ramify in layer 5. L, layer. (**B**) Resting membrane potentials (left), input resistances (middle), and capacitances (right) of *Ans*ACR^+ and *Ft*ACR^+ neurons. (**C**) Representative traces of light-evoked excitatory post-synaptic currents (EPSCs) recorded from ACR^- pyramidal neurons in layer 2/3 in response to 10-ms 470 nm light pulses (power density of 38.7 mW mm^{-2}). (**D**) Summary data of experiments in (C). The peak currents of light-evoked EPSCs were measured from the averaged current traces of three trials. In all panels, the data points from male mice are indicated by squares and female mice by circles. One male and one female mouse were used for each of the *Ans*ACR and *Ft*ACR experiments. Data are mean ± sem.

The online version of this article includes the following source data for Figure 6 – figure supplement 1:
Source data 1. Source data for the numbers of cells sampled and numerical values shown in (B, D).

bioRxiv preprint doi: https://doi.org/10.1101/2025.02.24.639930; this version posted March 1, 2025. The copyright holder for this preprint (which was not certified by peer review) is the author/funder, who has granted bioRxiv a license to display the preprint in perpetuity. It is made available under aCC-BY 4.0 International license.

Supplementary Table 1. Compositions and liquid junction potential (LJP) values of the solutions used in automated patch clamp recording.

	KF	NaCl	KCl	Na aspartate	$NaNO_3$	NMDG Cl	$CaCl_2$	$MgCl_2$	HEPES	EGTA	glucose	LJP
**Internal solution pH 7.2**	110	10	10				2	1	10	10		
**Standard external solution pH 7.4**		140	4				2	1	10		5	7.3
**Standard external solution pH 5.4**		140	4				2	1	10		5	7.3
**Na aspartate external solution**			4	140			2	1	10		5	-4
**$NaNO_3$ external solution**			4		140		2	1	10		5	6.4
**NMDG Cl external solution**			4			140	2	1	10		5	13.5
**KCl external solution**			144				2	1	10		5	2.6
